# The reference genome and full-length transcriptome of pakchoi provide insights into cuticle formation and heat adaption

**DOI:** 10.1093/hr/uhac123

**Published:** 2022-05-26

**Authors:** Huimin Xu, Chunhua Wang, Guirong Shao, Shasha Wu, Peng Liu, Ping Cao, Peng Jiang, Shubin Wang, Hong Zhu, Xiao Lin, Arfa Tauqeer, Yizhang Lin, Wei Chen, Weiqun Huang, Qingfang Wen, Jiang Chang, Fenglin Zhong, Shuang Wu

**Affiliations:** College of Life Sciences & College of Horticulture, Fujian Agriculture and Forestry University, Fuzhou 350002, China; College of Life Sciences & College of Horticulture, Fujian Agriculture and Forestry University, Fuzhou 350002, China; Fujian Jinpin Agricultural Technology Co., Ltd, Fuzhou 350000, China; College of Life Sciences & College of Horticulture, Fujian Agriculture and Forestry University, Fuzhou 350002, China; College of Life Sciences & College of Horticulture, Fujian Agriculture and Forestry University, Fuzhou 350002, China; Fujian Jinpin Agricultural Technology Co., Ltd, Fuzhou 350000, China; College of Life Sciences & College of Horticulture, Fujian Agriculture and Forestry University, Fuzhou 350002, China; College of Life Sciences & College of Horticulture, Fujian Agriculture and Forestry University, Fuzhou 350002, China; Fujian Seed Chief Station, Fuzhou 350003, China; Fujian Jinpin Agricultural Technology Co., Ltd, Fuzhou 350000, China; College of Life Sciences & College of Horticulture, Fujian Agriculture and Forestry University, Fuzhou 350002, China; Fujian Jinpin Agricultural Technology Co., Ltd, Fuzhou 350000, China; Fujian Seed Chief Station, Fuzhou 350003, China; Fujian Seed Chief Station, Fuzhou 350003, China; Crop Research Institute, Fujian Academy of Agricultural Sciences, Fuzhou 350013, China; College of Life Sciences & College of Horticulture, Fujian Agriculture and Forestry University, Fuzhou 350002, China; College of Life Sciences & College of Horticulture, Fujian Agriculture and Forestry University, Fuzhou 350002, China; College of Life Sciences & College of Horticulture, Fujian Agriculture and Forestry University, Fuzhou 350002, China

## Abstract

*Brassica rapa* includes various vegetables with high economic value. Among them, green petiole type pakchoi (*B. rapa* ssp. *chinensis*) is one of the major vegetables grown in southern China. Compared with other *B. rapa* varieties, green petiole type pakchoi shows a higher level of heat resistance, which is partially derived from the rich epicuticular wax. Here we sequence a high-quality genome of green petiole type pakchoi, which has been widely used as the parent in breeding. Our results reveal that long terminal repeat retrotransposon insertion plays critical roles in promoting the genome expansion and transcriptional diversity of pakchoi genes through preferential insertions, particularly in cuticle biosynthetic genes. After whole-genome triplication, over-retained pakchoi genes escape stringent selection pressure, and among them a set of cuticle-related genes are retained. Using bulked-segregant analysis of a heat-resistant pakchoi cultivar, we identify a frame-shift deletion across the third exon and the subsequent intron of *BrcCER1* in candidate regions*.* Using Nanopore long-read sequencing, we analyze the full-length transcriptome of two pakchoi cultivars with opposite sensitivity to high temperature. We find that the heat-resistant pakchoi cultivar can mitigate heat-caused leaf damage by activating an unfolded protein response, as well as by inhibiting chloroplast development and energy metabolism, which are presumably mediated by both transcriptional regulation and splicing factors. Our study provides valuable resources for *Brassica* functional genomics and breeding research, and deepens our understanding of plant stress resistance.

## Introduction

The *Brassica* genus comprises a variety of oilseed and vegetable crops [1, 2]. The ‘triangle of U’ model was proposed to describe the evolution of these *Brassica* species, including three diploid species, *Brassica rapa* (A genome), *B. nigra* (B genome), and *B. oleracea* (C genome), and three allopolyploid species, *Brassica napus* (A and C genome), *Brassica juncea* (A and B genome), and *Brassica carinata* (B and C genome) [3, 4]. The *Brassica* genomes have undergone three successive paleo-polyploidy events (γ, β, and α) [5, 6], followed by a Brassiceae-lineage-specific whole-genome triplication (WGT) [7–9]. This complex evolutionary history resulted in the asymmetric characteristics of the *Brassica* genomes [10].

*B. rapa* is a widely cultivated and economically important vegetable crop worldwide, which includes many subspecies, including Chinese cabbage, pakchoi, and turnip [11, 12]. Pakchoi is one of the most popular vegetables in Southeast Asia, and past natural selection and breeding past has allowed pakchoi to adapt to subtropical and tropical climates. Elucidation of the genomic basis of the adaptability of pakchoi would help us to understand the geographical distribution of this vegetable crop and accelerate future molecular breeding.

In addition to physiological adjustment, changes in organ or tissue characteristics can also affect plant responses to stresses. Cuticle, as a hydrophobic layer, has been known to play key roles in protection against excessive water evaporation, high temperature, UV radiation, and pathogen attack [13–15]. Compared with the closely related *B. rapa* subspecies, pakchoi forms relatively thicker cuticle layers, which probably enhance its adaptation to the subtropical and tropical climate in Southeast Asia. The previously published draft genomes of two *B. rapa* ssp*. chinensis* cultivars, purple pakchoi [16] and NHCC001 [17], have enhanced our understanding of *B. rapa* genome evolution. However, it is still not clear how natural selection and gene evolution have synergistically shaped pakchoi genome architecture and gene composition during long-term evolution and cultivation. In addition, breeding heat-resistant pakchoi cultivars has been hindered by the lack of gene resources, particularly heat-stress resistance (HSR) genes. A high-quality genome assembly, exploring pakchoi genome variation and mining agronomic trait-related genes, will greatly promote the genetic improvement of pakchoi.

Here, using single-molecule Nanopore long-read sequencing and high-throughput chromosome conformation capture (Hi-C) technologies, we constructed a high-quality genome assembly of the diploid genome of *B. rapa* ssp. *chinensis* cultivar PC-fu, a green petiole type pakchoi (2*n* = 2*x* = 20) that is rich in epicuticular wax and has high temperature resistance. In addition, we developed segregating populations of cultivars with opposite epicuticular wax traits, and identified the mutated site in *BrcCER1* by bulked sergeant analysis (BSA). Using Oxford Nanopore single-molecule sequencing, we analyzed the full-length transcriptome of the two cultivars with opposite sensitivity to high temperature. We found that both the gene expression pattern and the alternative splicing (AS) pattern play important roles in the high-temperature resistance and chloroplast stability of pakchoi. These results provide important resources for molecular breeding of pakchoi.

**Table 1:** Comparison between the PC-fu genome and other published *B. rapa* genome versions.

**Genome features**	**PC-fu**	**Purple pakchoi**	**NHCC001**	**Chiifu (v3)**
Genome assembly				
Assembled genome size (Mb)	411.4	370.4	405.3	353.1
GC content (%)	37.68		37.13	36.83
Number of scaffolds	2288		313	1301
Contig N50 (bp)	4700		3813	4437
Scaffold N50 (bp)	39 389	2820	2830	1498
TE rate (% of genome)	260.3 Mb (63.3%)	180.3 Mb (48.68%)	279.8 Mb (58.57%)	163.8 Mb (46.4%)
BUSCO				
Missing core genes (%)	0.80%	1.10%	0.93%	1.60%
Genome annotation				
Protein-coding genes	52 511	45 363	48 158	45 985
Average gene length (bp)	2220	1125	2119	1864
Reference	This study	[16]	[17]	[18]

## Results

### Genome assembly and annotation

We sequenced the genome of a commonly grown parent cultivar of green petiole type pakchoi, named ‘PC-fu’. Illumina short-read and single-molecule Nanopore long-read sequencing yielded ~36.70 Gb (73× genome coverage) and 60.69 Gb (147.52× genome coverage), respectively (Supplementary Data Table S1). The final assembly of 411 Mb was then generated, with 4.70 Mb for the contig N50 and 27.3 Mb for the longest contig (Table 1). Based on *k*-mer analysis of short-read sequences, the draft assembly accounts for ~80% of the estimated genome size of pakchoi (Supplementary Data Fig. S1). The completeness of the pakchoi genome assembly was further evaluated using Benchmarking Universal Single-Copy Ortholog (BUSCO) [19] and short-read mapping analysis. Our analysis showed that 97.5% of the total 1440 core eukaryotic genes were identified in the final assembly (Supplementary Data Table S2). For pakchoi the read alignment rate was high (>98%) and both tools indicated a meaningful improvement after the correction, suggesting final error rates were low at the base pair (bp) level. We also constructed Hi-C libraries of pakchoi. The result showed that 397.0 Mb (96.50%) of the assembly, including 1364 scaffolds, was placed on 10 linkage groups. Among them, the direction of 95.98% scaffolds could be determined in an orderly manner (Supplementary Data Fig. S2, Supplementary Data Table S3). A total of 52 511 protein-coding genes were annotated in the pakchoi genome using MAKER [19]. Of the predicted genes, 51 199 (97.5%) were homologous to at least one publicly known protein (Supplementary Data Table S4).

### Evolutionary analysis of *B. rapa* ssp. *chinensis*

The total of 533 single-copy orthologous genes from nine plant species were further identified to construct a maximum-likelihood phylogenetic tree (Supplementary Data Fig. S3A). It showed that *Brassica* diverged from the *Arabidopsis* lineage ~20 million years ago (MYA). Since then the *Brassica* lineage independently experienced WGT events ~14 MYA. Pakchoi (BRC) *and B. rapa* ssp. *pekinensis* (BRP) diverged at a relatively recent time of ~2.0 MYA (Supplementary Data Fig. S3A and S3B). We identified 24 979 orthologous gene families which originated from the most recent common ancestor. A total of 4882 species-specific genes belonging to 4771 gene family clusters were discovered in pakchoi.

To better understand the evolution of gene families during species divergence, we analyzed family expansion and contraction using CAFE software. The result indicated that 575 families comprising 6977 genes were significantly expanded (*P* < .001) and 44 families comprising 100 genes were considerably contracted (*P* < .001) in pakchoi. On the other hand, only 318 families (3164 genes) showed significant expansions (*P* < .001) and 174 families (450 genes) had contractions (*P* < .001) in *B. rapa* ssp. *pekinensis* after the divergence. In *Brassica*, multiple paralogous genes were retained in the triplicated synthetic regions that have promoted the evolution of the biosynthetic processes of primary metabolism [21]. Gene Ontology (GO) functional enrichment analysis of the significantly expanded gene families (*P* < .001) in pakchoi revealed that they are mainly involved in energy metabolism and other primary metabolisms associated with photosynthesis, oxidative phosphorylation, oxidation–reduction, and carbohydration (Supplementary Data Table S5, Supplementary Data Fig. S4).

We then annotated the functional domains of the expanded genes using the Pfam database and the SMART protein domain annotation resource, and found significant enrichment of the domains involved in the F-box associated domain, leucine-rich repeat (LRR), and protein kinase domain in pakchoi (*e*-value <.01) (Supplementary Data Fig. S5). F-box proteins play a wide range of roles in plant development, hormone signal transduction, and stress responses [22–25]. We identified 888 F-box coding genes in pakchoi, which is more than in *Arabidopsis thaliana* (692), *B. oleracea* (469), and eight other *B. rapa* subspecies [26] (Supplementary Data Fig. S6). F-box proteins can be classified into 19 categories based on C-terminal domains. Although the domain composition and organization of most F-box proteins were generally similar among these species, the larger categories, including LRR-FBD/LRR/FBD, FBA1/FBA3 and Kelch/Kelch repeats, showed significant differences in gene numbers (Supplementary Data Table S6). The phylogenetic analyses of all 2262 F-box genes from *A. thaliana* and two *B. rapa* subspecies showed that the three species shared 486 clusters, among which 210 clusters had similar gene number across species, indicating the intensive conservation of F-box genes. Thirty-three non-conservative clusters across the three species evolved into species-specific clusters. The clustered genes are mainly related to the processes of embryogenesis, male meiosis, photomorphogenesis, and stress responses [27, 28] (Supplementary Data Table S7). The evolutionary patterns of F-box genes seem to be associated with their functions.

### Genome expansion driven by long terminal repeat retrotransposons in pakchoi

**Figure 1:**
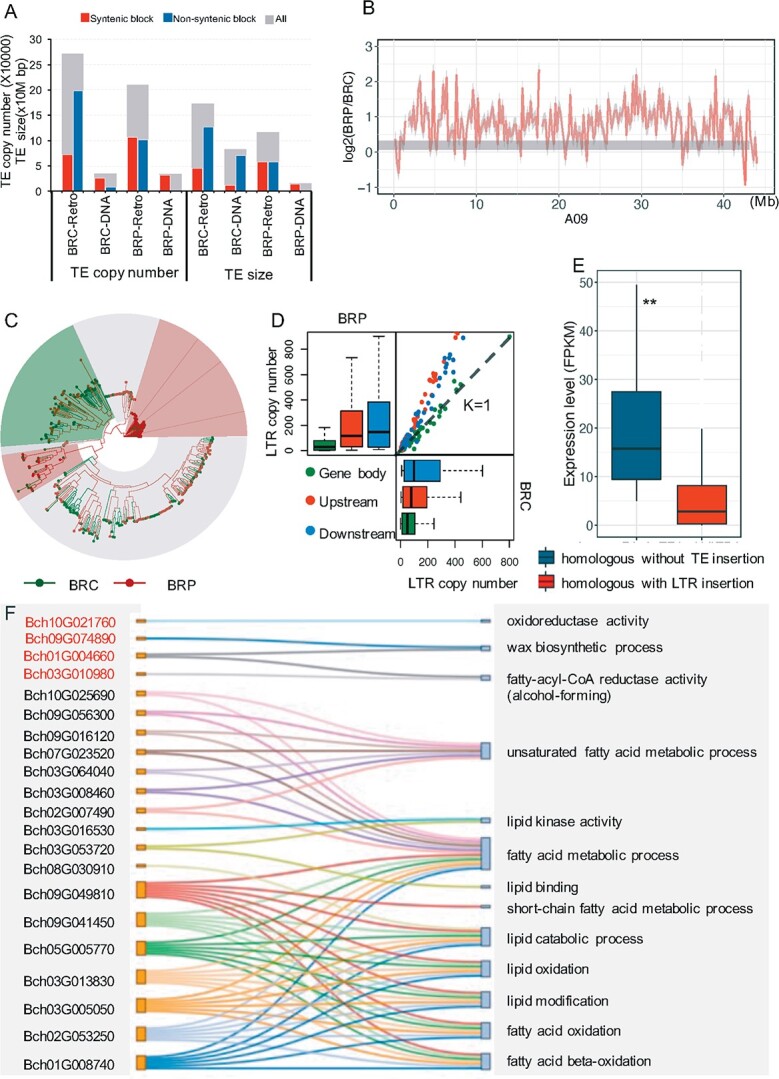
Comparison of TE distribution between pakchoi and Chinese cabbage*. (*A) TE copy number and total length in each genome and syntenic and non-syntenic regions. Retro, retrotransposon; DNA, DNA transposon. (B) Comparison of LTR distribution in pakchoi (BRC)–*B. rapa* ssp. *pekinensis* (BRP) syntenic blocks located in chromosome A09. (C) Phylogenetic tree of *Copia* as an example of intact LTRs in syntenic regions in BRC and BRP. (D) Copy number of LTRs located in the flanking regions (2 kb) and body region of the coding genes in the collinearity region in BRC and BRP. (E) Expression levels of homologous genes without TE insertion and with LTR insertion in BRC and BRP. ^**^P < .05. (F) GO terms related to cutin, suberin and wax biosynthesis (red font) among 670 genes.

**Figure 2:**
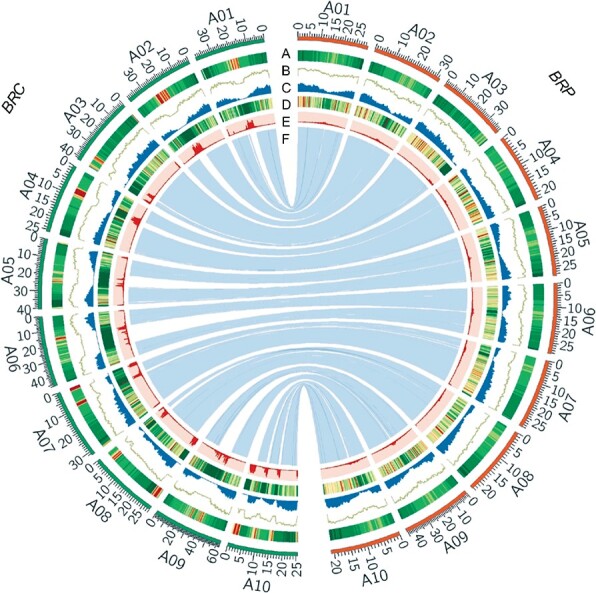
Characterization of chromosomes of the pakchoi (BRC) and Chinese cabbage (BRP) genomes. The outer layer of colored blocks represents the 20 pseudomolecules. Tracks are shown as follows: (A) TE density; (B) gene density; (C) A–X gene density; (D) gene expression; (E) LTR density; and (F) syntenic blocks.

Approximately 63.3% (260.3 Mb) of the pakchoi genome consisted of transposable elements (TEs), which is a larger proportion than in Chinese cabbage (163.8 Mb, 46.4%) (Table 1, Supplementary Data Table S8). Both DNA- (13.9 Mb) and retro- (172.1 Mb) TEs appeared to accumulate more in pakchoi compared with Chinese cabbage (15.4 Mb, 116.8 Mb)*.* To understand if this phenomenon is common in *B. rapa*, we further identified TEs in the genomes of seven additional representative *B. rapa* subspecies [26]. We found that the length of TEs in the pakchoi genome was slightly larger than that in six other subspecies (Supplementary Data Table S9). We next analyzed the distribution of TEs in syntenic and non-syntenic regions across nine genomes. The results showed that the length of retrotransposons in the syntenic region in pakchoi was slightly higher than that in other subspecies except for Chinese cabbage (Supplementary Data Fig. S7A). The length and numbers of long terminal repeat retrotransposon (LTR–RT) insertions in the non-syntenic blocks in pakchoi (126.3 Mb, 77.6%) were significantly higher than these in Chinese cabbage (57.8 Mb, 54.6%), but opposite trends were found in the syntenic region (Figs 1A and B and 2). Thus, It is a fact that Chinese cabbage has more retrotransposon insertions in the syntenic region than other subspecies.

As a result of the extensive accumulation of TEs, especially LTR–RTs in the non-syntenic regions, the genome size of pakchoi has become larger than that of Chinese cabbage*.* The phylogenetic analysis showed that Chinese cabbage had more LTR–RT families, and it had more members of most families (*Copia* and *Gypsy*) in the syntenic region than the pakchoi genome (Fig. 1C, Supplementary DataFig. S7B). Furthermore, in Chinese cabbage the total number of LTRs located in the flanking regions (2 kb) of the orthologous genes in the syntenic regions was significantly greater than that in pakchoi (Fig. 1D). As the insertion of LTR–RTs affects both genome structure and the transcription of the nearby genes, the LTRs in *B. rapa* subspecies could lead to the differential expression of these orthologous genes between Chinese cabbage and pakchoi. TE silencing is an important epigenetic suppression of gene expression, and transcriptionally inactivated genes can accumulate mutations, which eventually causes the inability to deregulate transposons [29–31]. To explore the functional importance of LTR–RT insertion, we compared the expression levels of homologous genes in the syntenic region, which include genes carrying at least one LTR insertion in Chinese cabbage while containing no adjacent TE insertion in pakchoi. The result revealed that the expression level of 670 homologous genes was significantly different in the two species*.* Compared with the genes with LTR insertions in Chinese cabbage, the corresponding homologous genes in pakchoi exhibited significantly higher expression levels (fold change >2, *P* < .05) (Fig. 1E). Combination of functional enrichment analyses by GO terms and Kyoto Encyclopedia of Genes and Genomes (KEGG) pathway mapping showed 670 genes are significantly enriched in multiple metabolisms, including arginine and proline metabolism (ko00330, ko00220), carotenoid biosynthesis (ko00906), and cutin/suberin/wax biosynthesis (ko00073) (*P* < .05) (Fig. 1F, Supplementary Data Table S10).

Covering the surface of most aerial organs, the cuticle is important in plant development and stress responses [32]. Among 670 genes, 4 are involved in cuticle biosynthesis, including three homologs of the *Arabidopsis ECERIFERUM 4* (*AtCER4*) gene, encoding alcohol-forming fatty acyl reductase (FAR), and one homolog of the *Arabidopsis ECERIFERUM 1* (*AtCER1*) gene encoding aldehyde decarbonylase. FAR enzymes catalyze the formation of primary alcohols, which act as the substrates for subsequent alkyl ester formation, while *CER1* is responsible for the conversion of stem wax long-chain (C30) aldehydes to alkanes (C29) (Supplementary Data Fig. S8). In *Arabidopsis*, *cer4* and *cer1* mutants exhibit reduction of wax [33, 34]. Interestingly, we found that a homologous gene pair, *Bch09g074890* and *BraA09g066480* (*CER1*), were highly expressed in pakchoi but silenced in Chinese cabbage. LTR insertion in the *CER1* gene of Chinese cabbage is located in the fourth intron, with a length of 461 bp. It was suggested that TE insertion of an intron often changes the alternative splicing pattern and potentially affects gene expression and evolution [35–37]. This differential expression pattern of wax genes caused by LTR–RTs presumably occurred after the split of subspecies of *B. rapa*, which could also have led to the higher accumulation of cuticle in pakchoi than in Chinese cabbage. This newly formed trait can help to reduce water loss [38–40], and may play a crucial role in the adaptation of pakchoi to the high temperatures of the subtropical environment.

**Figure 3:**
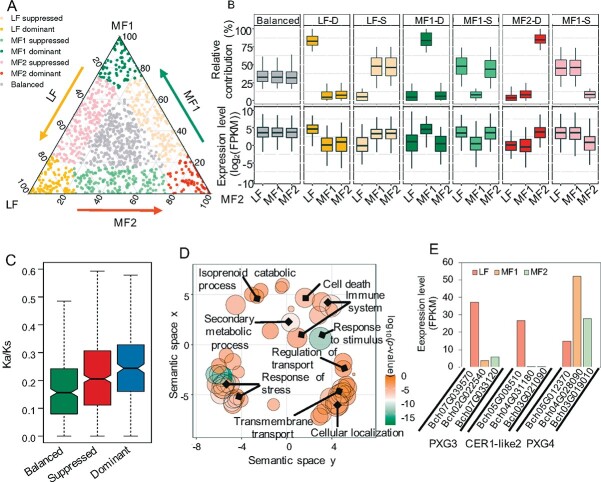
Expression bias in syntenic homolog triads. (A) Ternary plot showing the relative expression abundance of syntenic triads in three subgenomes. Each circle represents a gene triad. The coordinates composed of LF, MF1, and MF2 constitute the relative contribution of each homolog to the overall expression of the triad. (B) Relative contribution of each subgenome to the seven categories based on triad assignment, and the expression level of each subgenome in the seven categories. D, dominant; S, suppressed. (C) *K*_a_/*K*_s_ ratio for three homologs. (D) Top enriched GO terms of unbalanced triads. (E) Expression level of three triads involved in wax, cutin, and suberin biosynthesis.

### Performance of the dominant subgenome in environmental adaptation

A ‘two-step polyploidization’ model was proposed to explain the meso-triplication events in the origination of *B. rapa* species [10, 41]. During evolution, the MF1 and MF2 subgenomes (from two diploid genomes) experienced the first round of gene loss leading to a fractionated diploid genome [9, 41]. In addition, a third LF (another diploid genome) was added, and all three subgenomes experienced a second round of gene loss [9, 41]. To investigate how biased gene fractionation in the three subgenomes promotes the genome evolution of pakchoi, we constructed the ancestral karyotype (AK) blocks, which contain three copies derived from polyploidization and subsequent rearrangement (Supplementary Data Fig. S9, Supplementary Data Table S11). After polyploidization, duplicate-gene retention is complex and dynamic in gene evolution, including neo-functionalization, sub-functionalization and gene product dosage balance [42]. To functionally analyze the purifying selection of over-retained genes during the adaptive evolution of pakchoi, we identified 1581 1:1:1 correspondences across the three homologous subgenomes, referred to as triads. Among the 1581 triads, we identified 1197 syntenic triads that exhibited a high FPKM (fragments per kilobase of transcript per million mapped reads) value (>.5) for the summed expression of the three homologous subgenomes. The Euclidean distance and relative expression level were used to determine the position of syntenic triads in the ternary plot, which allowed us to define two groups of homology expression preference: balanced and unbalanced groups. The balanced one has no bias expression level among three homologs while the unbalanced one consists of six homolog-dominant or -suppressed groups (depending on whether the bias of one homolog has a higher or lower expression level than other two homologous genes) (Fig. 3A and B) [43]. Comparatively, the 343 syntenic triads belonging to the balanced group were fewer (28.7%), while syntenic triads classified as the unbalanced group were more frequent, consisting of 284 that were single-homolog-dominant (23.7%) and 570 that were single-homolog-suppressed (47.6%). The expression divergence among the subgenomes often leads to homolog neo- or sub-functionalization, which ultimately contributes to functional innovation in gene evolution [41, 43, 44]. Thus, we calculated the ratio of non-synonymous to synonymous substitutions (*K*_a_*/K*_s_) for three homologs, which was significantly higher for the unbalanced group than for the balanced group (*P* < .001, Fig. 3C). This result revealed that triads with expression divergence experienced more relaxed selection pressure than the triads with no-bias expression. Functional annotation of balanced triads demonstrated that they were mainly enriched in biological processes required for basic cellular functions (Supplementary Data Table S12). However, unbalanced groups were mainly enriched in secondary metabolic process, immune system, response to stimulus and stress (Fig. 3D). Interestingly, we found an enriched metabolic pathway involved in wax, cutin, and suberin biosynthesis in the unbalanced group, including three triads (nine genes) (ko00073, *P* < .05) of putative *PEROXYGENASE 3* (*PXG3*), *CER1-like 2*, and putative *PEROXYGENASE 4* (*PXG4*), which exhibited LF dominance or suppression (Fig. 3E). These findings support the idea that homologs with greater divergence in expression may lead to neo- or sub-functionalization after polyploidization. In pakchoi, over-retained genes escaped stringent selection pressure via the differentiation of gene expression to preserve duplicate genes. It was reported that several genes involved in the cuticle biosynthetic pathway had subgenome-preferred expression [45, 46], which is consistent with our results.

### Rapid mapping of cuticle-related loci in pakchoi using bulked segregant analysis (BSA)

**Figure 4:**
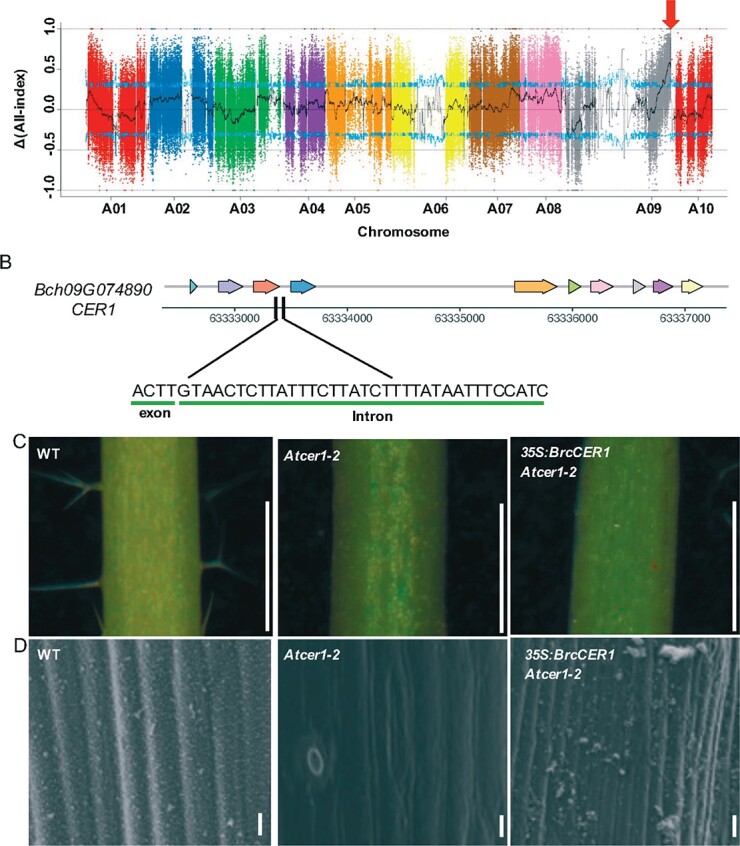
Identification of a candidate gene associated with epicuticular wax of pakchoi. (A) Identification of a candidate region associated with the waxy trait using SNP index association analysis. The *X*-axis represents the position of 10 chromosomes of pakchoi and the *Y*-axis represents the *Δ*(all-index). The color dots represent the *Δ*(all-index) value of every SNP locus. The black line shows the *Δ*(all-index) value of the fitting results. The blue lines represent the 95% confidence level. The red arrow indicates the causal mutation with high frequency. (B) Location of causal mutation of *BrcCER1*. JP1202 contains a frame-shift deletion across the third exon and the subsequent intron. (C) Epicuticular wax phenotype on the petiole of wild-type (WT), *Atcer1-2*, and *35S_pro_:BrcCER1-GFP/Atcer1-2* of *Arabidopsis*. Scale bar = 1 mm. (D) SEM analysis of epicuticular wax on the petiole of WT, *Atcer1-2*, and *35S_pro_:BrcCER1-GFP/Atcer1-2* at the rosette leaves stage. Scale bar = 10 μm.

To understand the genetics behind wax deposition in pakchoi, we examined two cultivars that exhibit opposite wax phenotypes: JP28, a glaucous cultivar containing obvious epicuticular wax deposition on leaves and petioles, and JP1202, a glossy cultivar containing little epicuticular wax on leaves and petioles (Supplementary Data Fig. S10A–C). Scanning electron microscopy (SEM) revealed a clear reduction of wax crystals in the leaf and petiole surface of JP1202 (Supplementary Data Fig. S10D). To identify the single-nucleotide polymorphisms (SNPs) behind the distinct phenotypes, we developed an *F*_2_ population by crossing JP28 and JP1202. A 3:1 ratio of JP28 to JP1202 plants was scored in the progeny, confirming that the glossy trait is likely conferred by a single recessive mutant gene. For BSA coupled with whole-genome sequencing (BSA-seq), we prepared four bulked pools, *F*_2_ plants with opposite cuticular wax traits, an S1 pool and an S2 pool (50 *F*_2_ plants for each), as well as an S3 pool and an S4 pool from two parents. AllWhole-genome resequencing with 50-fold depth was performed for all pools. We obtained 911 344 SNPs and 311 362 small indels between the two parents, including 59 839 non-synonymous SNPs. We further obtained 78 119 SNPs and 25 356 small indels that show a difference between the S1 and S2 pools, which included 2255 non-synonymous SNPs. The SNP index and indel index of the S1 and S2 pools were analyzed for markers across the whole genome, and their distribution on 10 chromosome was visualized with a 1-Mb interval using a 1-kb sliding window (Supplementary Data Fig. S11). The comparison of SNP index and indel index between the S1 and S2 pools, termed Δ(SNP index) and Δ(indel index), was further analyzed with a threshold at the 95% confidence level (Fig. 4A). Two candidate regions on chromosome 9 showed that *Δ*(all-index) [merged *Δ*(SNP-index) and *Δ*(indel-index)] was ≥1, suggesting they are the potential regions associated with the waxy loci (Supplementary Data Table S13). Within these candidate regions, there were 656 genes. We further identified 40 loci, among which all-index (merged SNP-index and indel-index) was 0 in the S1 pool and 1 in the S2 pool, as candidate polymorphism markers. Using ANNOVAR annotation, we identified the total of 22 candidate genes (Supplementary Data Table S14), including four genes with non-synonymous mutation, one gene with insertion, and one gene with deletion. Out of the 22 annotated genes, *Bch09G074890* is the putative candidate as its homolog *AtCER1* in *Arabidopsis* is involved in the metabolic pathway of cutin, suberin, and wax biosynthesis (Supplementary Data Fig. S8). We then compared the sequences of *Bch09G074890* from JP1202 and JP28. The result showed that *Bch09G074890* in JP1202 contains a frame-shift deletion across the third exon and the subsequent intron, which leads to early termination and disrupts the conservative FA_hydroxylase domain (Fig. 4B, Supplementary Data Fig. S12). We further examined the mutation of *Bch09G074890* in 17 additional collected cultivars, including 10 glaucous cultivars and 7 glossy cultivars. We found that the same mutation occurred in three glossy cultivars. Therefore, this causal mutation in *CER1* is strongly associated with wax deposition in pakchoi (Supplementary Data Fig. S13).

To verify the function of pakchoi *ECERIFERUM1 (BrcCER1)*, we constructed *35S_pro_:BrcCER1-GFP* and introduced it into *Arabidopsis* mutant *cer1-2* (SALK_014839), which shows a no-wax phenotype (Fig. 4C and D, Supplementary Data Fig. S14). The result showed that *BrcCER1* could complement the phenotype of *cer1-1* mutants (Fig. 4C and D), indicating that *BrcCER1* plays an important role in cuticular wax biosynthesis.

### Rapid and dynamic transcriptional regulation of pakchoi heat acclimation

**Figure 5:**
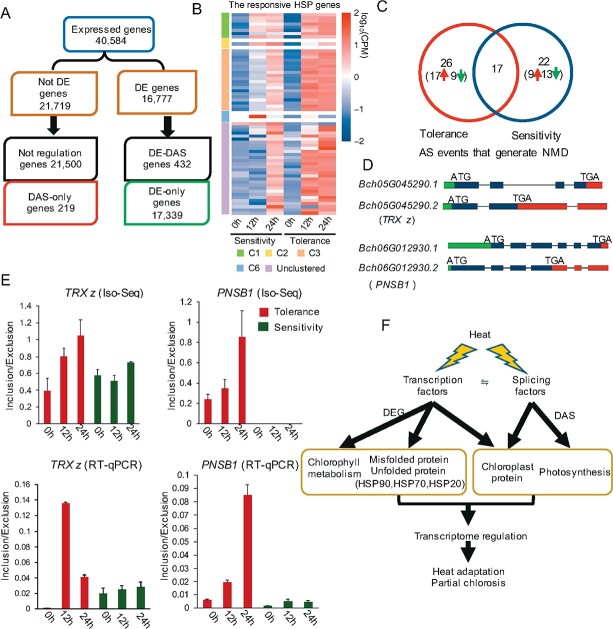
DE and DAS analyses of pakchoi response to heat stress. (A) Flow chart showing the distribution of the 17 771 DE and 650 DAS genes. The DE and DAS gene sets differ markedly, with only 432 genes in common. (B) Classification of gene expression heat map of responsive HSPs under heat stress. (C) Distribution of the responsive AS events that generate nonsense-mediated mRNA decay (NMD) in two cultivars. (D) Exon–intron structure of representative transcripts of four AS genes. (E) The colored bar graphs represent the inclusion/exclusion ratio of the IR events of DAS genes as identified from RNA-seq and RT–qPCR. (F) Model of the heat-responsive pathway and genome-wide transcriptional and post-transcriptional regulation.

To dissect the alterations in gene expression and AS under high temperature, we conducted full-length mRNA sequencing of the rosettes from two pakchoi cultivars—one of them (JP20) is sensitive while another (PC-fu) is tolerant to high temperature—using the single-molecule sequencing platform of Oxford Nanopore (Supplementary Data Fig. S15). Differential expression (DE) analysis revealed that a total of 16 777 genes [false discovery rate (FDR) <.01, |log_2_(fold change)| ≥2] were significantly affected by high temperature. Further, we identified 650 differential alternative splicing (DAS) genes (882 DAS events) [genes with variation in the percentage splicing index (PSI) of AS events in different conditions exceeding the threshold (FDR <.01, *Δ*PSI ≥10%) are regarded as DAS genes] (Fig. 5A), of which 432 were also DE genes (regulated by both transcription and AS) and 218 genes were only regulated by AS (Supplementary Data Tables S15 and S16). We then classified all DE genes into 10 clusters with unique expression patterns (C1-C10) (FDR <.05, |fold change| ≥2) (Supplementary Data Fig. S16). Among the 10 clusters, C1–C6 represent upregulated genes induced by high temperature. In contrast, the expression patterns of C7–C10 showed downregulation after heat stress. Clusters C1–C6 were significantly enriched in known GO terms of physiological and molecular events that are related to heat response (Supplementary Data Fig. S16). Interestingly, the most significant GO enrichment term in cluster C3 was ‘protein folding’ (GO:0006457), most of the proteins endcode by those genes in this category the heat-shock proteins (HSPs). HSPs play an essential role in the maturation of protein complexes and the degradation of damaged or misfolded peptides [47]. Our result showed that the transcriptional level of HSPs in the tolerant cultivar was higher than that in the sensitive cultivar under heat stress (Fig. 5B). In addition, HSPs that were not clustered in response to high temperature were rapidly upregulated in the tolerant cultivar. These findings suggest that the tolerant cultivar likely has stronger ability to repair damaged proteins than the sensitive cultivar under heat stress.

Other genes that were downregulated by heat were mainly involved in chlorophyll synthesis, chloroplast and thylakoid membrane and components, as well as amino acid metabolism (Supplementary Data Fig. S16). Consistent with the leaf chlorosis phenotypes, the genes involved in the chloroplast synthesis pathway were downregulated in both tolerant and sensitive cultivars under heat stress (Supplementary Data Fig. S17). These results suggest that pakchoi copes with heat stress presumably through both repressing chloroplast development and repairing misfolded peptides as well as stabilizing organelle structure.

Other most enriched functional terms for DAS genes were related to mRNA splicing, spliceosomal complex, and chloroplast. We performed different transcript usage (DTU) analysis to ascertain the individual transcript responsible for DAS genes. In total, 35.27% (432/1225) of expressed transcripts of DAS genes were classed as DTU (FDR <.01, *Δ*PSI >0.1; see Materials and methods). Hierarchical clustering of the DTU transcripts and expression patterns of individual DAS genes also exhibited transient, adaptive, and late expression patterns (Supplementary Data Fig. S18). Functional annotation indicated the enrichment of terms in the chloroplast. Thus, we speculated that the expression patterns and AS patterns of these genes reflect the different contributions to high temperature resistance.

Most DAS genes related to chloroplast were also DE genes (100 out of 131), suggesting chloroplast genes are regulated by both transcript and AS under heat stress. Furthermore, under heat stress, 163 DAS events were defined from the 131 genes and 39.9% of DAS (65) generated 74 transcripts (isoform) (Fig. 5C). These DAS events introduced premature termination codons, resulting in truncated proteins or nonsense-mediated mRNA decay (NMD). We also identified a high frequency of intron retention (IR) (46%) in both cultivars (Supplementary Data Fig. S19). To evaluate the effect of heat-stress induced AS on chloroplast, we investigated the AS isoforms and the structure of chloroplast genes. For example, *Bch05G045290*), an ortholog of *Arabidopsis RAP10*, encodes THIOREDOXIN Z (*TRX z*). *TRX z* was shown to interact with fructokinase-like protein (FLN) to form a TRX–FLN regulatory module to regulate the transcription of the PEP-encoded (PEP, plastid-encoded RNA polymerase) genes and maintain the redox balance in chloroplasts under heat stress [48–50]. By contrast, heat stress conditions induced the retention of the second intron in the transcripts of *BrcTRX z*, resulting in a truncated thioredoxin (TRX)-like domain in the tolerant cultivar (Fig. 5D and E). The NAD(P)H dehydrogenase (NDH) complex functions in photosystem I cyclic electron transfer and is vital for abiotic stress responses in plants [51, 52]. We further analyzed a gene, photosynthetic NDH subcomplex B1 (*PNSB1*) (*Bch06G012930*), which is involved in the cyclic electron flow of photosystem I and produces ATP. We found that heat stress considerably affected the ratio of IR of the third intron in this gene, producing a truncated protein containing only a RfaF domain in the tolerant cultivar (Fig. 5D and E).

The fluctuations of DE and AS in response to heat reflect regulation by transcription factors (TFs) and SF/RNA binding proteins (SF-RBPs). Here, 1329 TFs were significantly regulated at transcriptional level, while only 37 TFs were DAS and 27 TFs were DE + DAS. The predicted SF-RBP genes included 371 DE-only genes, 19 DAS-only genes, and 18 DE + DAS genes. The TF genes had a high proportion of stress/hormone response genes and genes associated with organ developmental processes (Supplementary Data Table S17). The SF-RBP genes contained serine/arginine-rich (SR), chloroplast ribonucleoprotein particle (RNP) and chloroplast splicing protein genes such as *SCL30A*, *SR34A*, *CP29*, and *CRS1* [53–55] (Supplementary Data Table S18). Both transcriptional and post-transcriptional regulations of mRNA determine the expression levels of TFs and SFs. Among them, many genes were rapidly regulated in response to heat stress (Fig. 5F).

In summary, the splicing of chloroplast related genes is changed by the reduced removal rate of introns under heat stress, which often introduces premature termination codons. This additional AS regulation presumably protects chloroplasts and reduces energy transport to enhance heat acclimatization of pakchoi. The functional significance of AS regulation under heat stress could be greater than previously thought, and it provides an additional regulatory layer to help plant adaptation to higher temperatures.

## Discussion

Pakchoi is one of the economically important vegetables in Asia, and it provides an excellent model for the genetical study of the phenotypic diversity in *Brassica* plants. The current draft genome of pakchoi adds to the growing body of genome information for the *Brassica* genus. *B. rapa* has experienced WGT events, and as a result, several genes have paralogs in syntenic blocks. Many external environmental factors promote the production of polyploids [56, 57]. Therefore, duplicated genes are the products of the ecosystem and are constantly affected by environmental changes.

A large number of repetitive sequences is an attractive feature of the genome of *Brassica* plants. Our results suggested that the accumulation of TE plus large numbers of LTRs enlarged the genome size of pakchoi, making it larger than that of Chinese cabbage. These genome level changes also drove the functional diversity of certain homologous genes in the two *B. rapa* subspecies. Interestingly, there are more LTRs in pakchoi that ony occurs in the non-collinearity region (centromere region) than in Chinese cabbage. In contrast, the number and length of LTRs located in the flanking regions (2 kb) and the body region of the Chinese cabbage genes are greater than those of homologous genes in the collinearity region of pakchoi. As a result, the expression level of LTR-disturbed genes in the collinearity region of Chinese cabbage is reduced, while the corresponding homologous genes in pakchoi contain no TE insertion and thus have higher expression levels. Four of these genes are involved in cutin, suberin, and wax biosynthesis. For example, *Bch09g074890* and *Bra09g066480* (*CER1*) show high expression in pakchoi but are silenced in Chinese cabbage. These results support the idea that LTRs drive the formation of plant functional centromeres in heterochromatin around the centromere [58, 59]. Moreover, LTR insertion not only affects gene and genome structure, thereby increasing genome instability, but also affects the expression of nearby genes, thus affecting phenotypic varieties across two subspecies during genome evolution [60–62].

The pakchoi sequenced in this study have two characteristics: rich epicuticular wax and high temperature resistance. Under a variety of abiotic and biotic stresses, the cuticle wax acts as a protective barrier. Planted in Southeast Asia, pakchoi has strong water-holding capacity and high temperature resistance. Our results in this study explain how the cuticle-related genes escaped selection pressure during evolution. In addition, we identified candidate genes associated with the epicuticular wax trait using BSA analysis, which could be used as a morphological marker in hybrid breeding in *Brassica*. In addition to JP28 and JP1202, the same mutation event (a frame-shift deletion across the third exon and the subsequent intron of *BrcCER1*) was detected in three other glossy cultivars. For the remaining four glossy varieties without this mutation, we speculate that different mutations in other genes or loci could exist.

Transcriptional changes and post-transcriptional changes generated by AS play important roles in heat stress resistance. In this study, we found that two pakchoi cultivars with opposite sensitivity to high temperature showed different degrees of heat-induced chlorosis. Consistent with this phenotype, the expression level of HSPs was alternated more dramatically in the pakchoi cultivar that is tolerant to heat than the sensitive cultivar. More importantly, heat stress inhibits the production of chloroplast proteins not only by suppressed transcription, but also by AS. For example, the retention of introns of chloroplast-related genes, often occurring under heat stress, can cause early termination and truncated proteins. The AS regulation mechanism protects chloroplasts and reduces energy transport to enhance heat acclimatization. Interestingly, AS-induced NMD for chloroplast-related genes has occurred more in the tolerant cultivar than in the sensitive cultivar under heat stress.

In summary, our results provide a comprehensive resource for genetic improvement of important traits in *Brassica*, including the formation of epicuticular wax and high temperature resistance. This is also crucial for improving agricultural breeding under the threat of climate change.

## Materials and methods

### Sample preparation and genome sequencing

The pakchoi cultivar PC-fu was collected in Fujian Province of China. Expanded and fresh leaves were collected and immediately frozen in liquid nitrogen. The tissues were stored at −80°C in the laboratory before DNA extraction. High-quality genomic DNA was extracted for Nanopore and Illumina sequencing. High-quality data (36.70 GB) were obtained and screened on the Illumina Hiseq sequencing platform with Q20 (%) >98.05 and Q30 (%) >94.65. The read quality value of the raw data sequenced on the Nanopore sequencing platform was preliminarily filtered, and reads with low quality and short length (2 kb) were filtered out. According to statistics, the read N50 is 41 135 bp and the mean read length is 29 338 bp.

### Genome assembly and evaluation of assembly quality

After filtering the raw nanopore data for low quality and short clips, we performed initial read correction using Canu software [63] and assembled the corrected reads using WTDBG2 (https://github.com/ruanjue/wtdbg) and SMARTdenovo software (https://github.com/ruanjue/smartdenovo). Racon software [64] was used to correct consensus assembly. To obtain the final assembly, we polished the Illumina read data with Pilon software [65]. The statistical information is shown in Supplementary Data Table S1. The assembly quality was assessed using BUSCO v4.0.2 with Embryophyta OrthoDB v9. We mapped Illumina reads against the polished assembly using BWA-MEM [66] with default parameters to estimate the completeness of the genome assembly. We anchored 1364 scaffolds to 10 pseudo-molecules by employing LACHESIS based on Hi-C data [67].

### Gene prediction and functional annotation

To predict protein-coding genes of the pakchoi genome, we used three strategies, including *ab initio*, homology, and RNA-seq based prediction. To annotate the function of the protein-coding genes, we aligned these genes to the NCBI non-redundant protein sequences (NR) [68], Eukaryotic Orthologous Groups of Proteins (KOG) [69], KEGG [70] and TrEMBL [71] databases using BLAST (v2.2.31) [72]. GO [73, 74] annotations were executed by Blast2GO (v4.1) [75]. We also annotated genes encoding transcription factors using iTAK programmer (v18.12) [76]. The putative domains of proteins were identified using SMART [77] and HMMER [78] based on the PFAM database [79].

### Repeat annotation

RepeatModeler (v4.1.0) [80] was used to develop a *de novo* repeat library, and the repeat families were identified by RECON (v1.05) and RepeatScout (v1.0.6) The database was classified by PASTEClassifier [81], and then merged with the RepBase library to form the final repetitive sequence database. RepeatModeler was used to evaluate repeat copies, proportion, and distribution in the pakchoi genome.

Intact LTR–RTs were identified in pakchoi and Chinese cabbage genome assemblies using LTR_harvest [82]. The outputs (.scn) for the two above species were integrated by LTR retriever software [83]. LTR–RT sequences of *Copia* and *Gypsy* were aligned separately by MAFFT (v7.158) [84], and the tree was generated using FastTree (v1.0.36) [85].

### Gene families and phylogenetic analysis

Gene families/clusters in the pakchoi genome and genomes of other species were identified using the OrthoFinder package (v2.2.7) [86]. We investigated the expansion and contraction of gene families using CAFE software (v.2.1) [87]. Phylogenetic relationship was resolved using MAFFT [84] and the FastTree package [85]. Divergence times of species were predicted by the r8s program [88].

### Analysis of genome synteny and whole-genome duplication

We identified whole-genome duplication events by searching for collinearity within the pakchoi genome or between pakchoi and other closely related species genomes using MCScan v0.8 software [89]. We estimated *K*_s_ and *K*_a_ for gene pairs within the collinear segment using synonymous_calculation (https://github.com/tanghaibao/bio-pipeline/tree/master/synonymous_calculation).

### RNA-seq analysis

We filtered raw RNA sequencing reads using fastp software to remove low-quality bases, adaptors, duplications, and potential contaminations [90]. We next mapped clean reads to the assembled pakchoi genome using the HISAT2 program with default settings [91]. Gene expression level was calculated using Cufflinks [92] and quantified by FPKM.

### BSA-seq analysis

Based on the locating data of clean reads in the reference genome, we removed duplicate reads using the Picard tool (http://sourceforge.net/projects/picard/). The SNPs between the test samples and the assembled pakchoi genome were obtained using the GATK program (v4.1.4.1) [93].

We calculated the SNP index value to identify the candidate regions associated with waxy in the genome. The *△*(SNP-index), *△*(indel-index) and *△*(all-index) of each locus was calculated by subtraction of the value of the S1 pool from that of the S2 pool. The corresponding *△*(SNP-index), *△*(indel-index), and *△*(all-index) of each marker locus in each window were counted by the sliding window method. The windows above the confidence level (95%) were regarded as candidate regions. ANNOVAR [94] was used to annotate the candidate polymorphic markers.

### Full-length transcriptome sequencing and analysis under heat stress

Full-length transcriptome data reported in this paper were obtained using Nanopore sequencing. We identified full-length non-chimeric (FLNC) transcripts by searching for primers at both ends of reads. Clusters of FLNC transcripts were then obtained after being mapped to the reference genome with minimap2 [95]. Consensus isoforms were identified after cleaning within each cluster by pinfish, and they were then mapped to the genome by minimap2. The above mapped reads were collapsed by the cDNA_Cupcake package (https://github.com/Magdoll/cDNA_Cupcake/wiki).

Differential expression genes (DEGs) were identified using the DESeq package [96]. Genes/transcripts with FDR <.01 and fold change ≥2 were designated as DEGs. Gene expression pattern analysis of tolerance/sensitivity cultivars was executed using Short Time-series Expression Miner software (STEM) [97] on the OmicShare tools platform (www.omicshare.com/tools).

DAS and DTU analysis was performed using SUPPA [98]. Genes/transcripts with significant DAS or DTU had adjusted FDR <.01 for at least two contrast groups, and these contrast groups had at least ≥10% change in PSI.

An additional file with more details is shown in [Supplementary files].

## Acknowledgements

This study was supported by National Key Programs for Bok Choy Breeding of China (11821301354052283-2), the Provincial Natural Science Foundation of Fujian (2019 J01422) and the National Natural Science Foundation of China (31900169).

## Author contributions

S.W., H.X., C.W., and F.Z designed this study; H.X., C.W., P.L., S.W., W.C., W.H., and Y.L. contributed to the sample preparation and sequencing; H.X., C.W., and P.L performed genome assembly and annotation; H.X., C.W., P.J., A.T. and J.C performed comparative genomic analysis. F.Z., C.W., S.W., X.L., Q.W., and H.Z. processed BSA data; C.W., H.X., P.L., P.C., G.S., and S.W. processed high-temperature data. S.W., H.X., and C.W. wrote and revised the manuscript.

## Data availability

Raw sequencing reads in the present study have been submitted to the National Center for Biotechnology Information bioproject database (accession number PRJNA735552). The genome assembly, gene annotation and gene expression data are available on the figshare database (https://doi.org/10.6084/m9.figshare.19589524.v2).

## Conflict of interest

All authors confirm that they have no conflict of interest.

## Supplementary data

Supplementary data is available at *Horticulture Research* online.

## Supplementary Material

Suppl_uhac123

## References

[1] Warwick SI, Francis A, Al-Shehbaz IA. Brassicaceae: species checklist and database on CD-rom. Plant Syst and Evol. 2006;259:249–25.

[2] Cheng F, Wu J, Wang X. Genome triplication drove the diversification of *Brassica* plants. *Hortic Res*. 2014;1:14024. 26504539 10.1038/hortres.2014.24 PMC4596316

[3] Song X, Wei Y, Xiao D et al. *Brassica carinata* genome characterization clarifies U's triangle model of evolution and polyploidy in *Brassica*. *Plant Physiol*. 2021;186:388–406. 33599732 10.1093/plphys/kiab048 PMC8154070

[4] Nagaharu U . Genome analysis in brassica with special reference to the experimental formation of *B. napus* and peculiar mode of fertilization. *Jpn J Bot*. 1935;7:389–452.

[5] Bowers JE, Chapman BA, Rong J et al. Unravelling angiosperm genome evolution by phylogenetic analysis of chromosomal duplication events. *Nature*. 2003;422:433–8. 12660784 10.1038/nature01521

[6] Jiao Y, Wickett NJ, Ayyampalayam S et al. Ancestral polyploidy in seed plants and angiosperms. *Nature*. 2011;473:97–100. 21478875 10.1038/nature09916

[7] Yang YW, Lai KN, Tai PY et al. Rates of nucleotide substitution in angiosperm mitochondrial DNA sequences and dates of divergence between *Brassica* and other angiosperm lineages. *J Mol Evol*. 1999;48:597–604. 10198125 10.1007/pl00006502

[8] Town CD, Cheung F, Maiti R et al. Comparative genomics of *Brassica oleracea* and *Arabidopsis thaliana* reveal gene loss, fragmentation, and dispersal after polyploidy. *Plant Cell*. 2006;18:1348–59. 16632643 10.1105/tpc.106.041665 PMC1475499

[9] Wang X, Wang H, Wang J et al. The genome of the mesopolyploid crop species *Brassica rapa*. *Nat Genet*. 2011;43:1035–9. 21873998 10.1038/ng.919

[10] Cheng F, Mandáková T, Wu J et al. Deciphering the diploid ancestral genome of the mesohexaploid *Brassica rapa*. *Plant Cell*. 2013;25:1541–54. 23653472 10.1105/tpc.113.110486 PMC3694691

[11] Zhao J, Wang X, Deng B et al. Genetic relationships within *Brassica rapa* as inferred from AFLP fingerprints. *Theor Appl Genet*. 2005;110:1301–14. 15806345 10.1007/s00122-005-1967-y

[12] Xiao D, Wang H, Basnet RK et al. Genetic dissection of leaf development in *Brassica rapa* using a genetical genomics approach. *Plant Physiol*. 2014;164:1309–25. 24394778 10.1104/pp.113.227348 PMC3938622

[13] Zhang X, Liu Z, Wang P et al. Fine mapping of BrWax1, a gene controlling cuticular wax biosynthesis in Chinese cabbage (*Brassica rapa* L. ssp. *pekinensis*). *Mol Breed*. 2013;32:867–74.

[14] Sturaro M, Motto M, Hemantaranjan A. Plant cuticular waxes: biosynthesis and functions. *Adv Plant Physiol*ogy. 2006;9:229–51.

[15] Pu Y, Gao J, Guo Y et al. A novel dominant glossy mutation causes suppression of wax biosynthesis pathway and deficiency of cuticular wax in *Brassica napus*. *Plant Biol*. 2013;13:215–5. 10.1186/1471-2229-13-215 PMC3881019 24330756

[16] Li P, Su T, Zhao X et al. Assembly of the non-heading pak choi genome and comparison with the genomes of heading Chinese cabbage and the oilseed yellow sarson. *Plant Biotechnol J*. 2021;19:966–76. 33283404 10.1111/pbi.13522 PMC8131043

[17] Li Y, Liu GF, Ma LM et al. A chromosome-level reference genome of non-heading Chinese cabbage [*Brassica campestris* (syn. *Brassica rapa*) ssp. *chinensis*]. Hortic Res 2020;7:212 10.1038/s41438-020-00449-z PMC7769993 33372175

[18] Zhang L et al. Improved Brassica rapa reference genome by single-molecule sequencing and chromosome conformation capture technologies. *Hortic Res*. 2018;5:50. 30131865 10.1038/s41438-018-0071-9 PMC6092429

[19] Simão FA, Waterhouse RM, Ioannidis P et al. BUSCO: assessing genome assembly and annotation completeness with single-copy orthologs. *Bioinformatics*. 2015;31:3210–2. 26059717 10.1093/bioinformatics/btv351

[20] Campbell MS, Holt C, Moore B et al. Genome annotation and curation using MAKER and MAKER-P. *Curr Protoc Bioinformatics*. 2014;48: 4.11.1-39. 10.1002/0471250953.bi0411s48 PMC4286374 25501943

[21] Liu S, Liu Y, Yang X et al. The *Brassica oleracea* genome reveals the asymmetrical evolution of polyploid genomes. *Nat Commun*. 2014;5:3930. 24852848 10.1038/ncomms4930 PMC4279128

[22] Zheng N, Schulman BA, Song L et al. Structure of the Cul1–Rbx1–Skp1–F box^Skp2^ SCF ubiquitin ligase complex. *Nature*. 2002;416:703–9. 11961546 10.1038/416703a

[23] Xu G, Ma H, Nei M et al. Evolution of F-box genes in plants: different modes of sequence divergence and their relationships with functional diversification. Proc Natl Acad Sci *USA* 2009;106:835–40. 19126682 10.1073/pnas.0812043106 PMC2630105

[24] Calderón-Villalobos LIA, Nill C, Marrocco K et al. The evolutionarily conserved *Arabidopsis thaliana* F-box protein AtFBP7 is required for efficient translation during temperature stress. *Gene*. 2007;392:106–16. 17240087 10.1016/j.gene.2006.11.016

[25] Calderón Villalobos LIA, Lee S, de Oliveira C et al. A combinatorial TIR1/AFB–aux/IAA co-receptor system for differential sensing of auxin. *Nat Chem Biol*. 2012;8:477–85. 22466420 10.1038/nchembio.926 PMC3331960

[26] Cai X, Chang L, Zhang T et al. Impacts of allopolyploidization and structural variation on intraspecific diversification in *Brassica rapa*. *Genome Biol*. 2021;22:166. 34059118 10.1186/s13059-021-02383-2 PMC8166115

[27] Kuroda H, Yanagawa Y, Takahashi N et al. A comprehensive analysis of interaction and localization of *Arabidopsis* SKP1-LIKE (ASK) and F-box (FBX) proteins. *PLoS One*. 2012;7:e50009. 23166809 10.1371/journal.pone.0050009 PMC3499479

[28] Zhang Y, Xu W, Li Z et al. F-box protein DOR functions as a novel inhibitory factor for abscisic acid-induced stomatal closure under drought stress in *Arabidopsis*. *Plant Physiol*. 2008;148:2121–33. 18835996 10.1104/pp.108.126912 PMC2593669

[29] Pélissier T, Mathieu O. Glue for jumping elements: epigenetic means for controlling transposable elements in plants. In: Grandbastien M-A, Casacuberta JM, eds. Plant Transposable Elements: Impact on Genome Structure and Function. Springer: Berlin, 2012,125–45.

[30] Lisch D . How important are transposons for plant evolution? *Nature Rev Genet*. 2013;14:49–61. 23247435 10.1038/nrg3374

[31] Feschotte C . Transposable elements and the evolution of regulatory networks. *Nat Rev Genet*. 2008;9:397–405. 18368054 10.1038/nrg2337 PMC2596197

[32] Bernard A, Joubès JM. *Arabidopsis* cuticular waxes: advances in synthesis, export and regulation. *Prog Lipid Res*. 2013;52:110–29. 23103356 10.1016/j.plipres.2012.10.002

[33] Bourdenx B, Bernard A, Domergue F et al. Overexpression of *Arabidopsis* ECERIFERUM1 promotes wax very-long-chain alkane biosynthesis and influences plant response to biotic and abiotic stresses. *Plant Physiol*. 2011;156:29–45. 21386033 10.1104/pp.111.172320 PMC3091054

[34] Owen Rowland HZ, Hepworth SR, Lam P et al. CER4 encodes an alcohol-forming fatty acyl-coenzyme a reductase involved in cuticular wax production in *Arabidopsis*. *Plant Physiol*. 2006;142:866–77. 16980563 10.1104/pp.106.086785 PMC1630741

[35] Hickey DA, Benkel B. Introns as relict retrotransposons: implications for the evolutionary origin of eukaryotic mRNA splicing mechanisms. *J Theor Biol*. 1986;121:283–91. 3025526 10.1016/s0022-5193(86)80108-4

[36] Jaligot E, Hooi WY, Debladis E et al. DNA methylation and expression of the EgDEF1 gene and neighboring retrotransposons in mantled somaclonal variants of oil palm. *PLoS One*. 2014;9:e91896. 24638102 10.1371/journal.pone.0091896 PMC3956824

[37] Galindo-González L, Mhiri C, Deyholos MK et al. LTR-retrotransposons in plants: engines of evolution. *Gene*. 2017;626:14–25. 28476688 10.1016/j.gene.2017.04.051

[38] Bhanot V, Fadanavis SV, Panwar J. Revisiting the architecture, biosynthesis and functional aspects of the plant cuticle: there is more scope. *Environ Exp Bot*. 2021;183:104364.

[39] Bueno A, Alfarhan A, Arand K et al. Effects of temperature on the cuticular transpiration barrier of two desert plants with water-spender and water-saver strategies. *J Exp Bot*. 2019;70:1613–25. 30715440 10.1093/jxb/erz018 PMC6416792

[40] Fedoroff NV, Battisti DS, Beachy RN et al. Radically rethinking agriculture for the 21st century. *Science*. 2010;327:833–4. 20150494 10.1126/science.1186834 PMC3137512

[41] Cheng F, Wu J, Fang L et al. Biased gene fractionation and dominant gene expression among the subgenomes of *Brassica rapa*. *PLoS One*. 2012;7:e36442. 22567157 10.1371/journal.pone.0036442 PMC3342247

[42] Krishnamurthy P, Kim JA, Jeong MJ et al. Gene loss/retention and evolutionary pattern of ascorbic acid biosynthesis and recycling genes in *Brassica rapa* following whole genome triplication. *Genes Genomics*. 2016;38:1129–43.

[43] Ramírez-González RH, Borrill P, Lang D et al. The transcriptional landscape of polyploid wheat. *Science*. 2018;361:eaar6089. 30115782 10.1126/science.aar6089

[44] Schnable JC, Springer NM, Freeling M. Differentiation of the maize subgenomes by genome dominance and both ancient and ongoing gene loss. Proc Natl Acad Sci *USA* 2011;108:4069–74. 21368132 10.1073/pnas.1101368108 PMC3053962

[45] Bergh EVD, Hofberger JA, Schranz ME. Flower power and the mustard bomb: comparative analysis of gene and genome duplications in glucosinolate biosynthetic pathway evolution in Cleomaceae and Brassicaceae. *Am J Bot*. 2016;103:1212–22. 27313198 10.3732/ajb.1500445

[46] Weike D, Song X, Liu T et al. Patterns of evolutionary conservation of ascorbic acid-related genes following whole-genome triplication in *Brassica rapa*. *Genome Biol Evol*. 2014;7:299–313. 25552535 10.1093/gbe/evu293 PMC4316640

[47] Pratt WB, Toft DO. Regulation of signaling protein function and trafficking by the hsp90/hsp70-based chaperone machinery. *Exp Biol Med (Maywood)*. 2003;228:111–33. 12563018 10.1177/153537020322800201

[48] Arsova B, Hoja U, Wimmelbacher M et al. Plastidial thioredoxin z interacts with two fructokinase-like proteins in a thiol-dependent manner: evidence for an essential role in chloroplast development in *Arabidopsis* and *Nicotiana benthamiana*. *Plant Cell*. 2010;22:1498–515. 20511297 10.1105/tpc.109.071001 PMC2899873

[49] Wimmelbacher M, Börnke F. Redox activity of thioredoxin z and fructokinase-like protein 1 is dispensable for autotrophic growth of *Arabidopsis thaliana*. *J Exp Bot*. 2014;65:2405–13. 24659486 10.1093/jxb/eru122 PMC4036507

[50] Lv Y, Shao G, Qiu J et al. White leaf and panicle 2, encoding a PEP-associated protein, is required for chloroplast biogenesis under heat stress in rice. *J Exp Bot*. 2017;68:5147–60. 29045742 10.1093/jxb/erx332 PMC5853965

[51] Sirpiö S, Allahverdiyeva Y, Holmström M et al. Novel nuclear-encoded subunits of the chloroplast NAD(P)H dehydrogenase complex. *J Biol Chem*. 2009;284:905–12. 18974055 10.1074/jbc.M805404200

[52] Sun Y, Geng Q, Du Y et al. Induction of cyclic electron flow around photosystem I during heat stress in grape leaves. *Plant Sci*. 2017;256:65–71. 28167040 10.1016/j.plantsci.2016.12.004

[53] Wang S, Bai G, Wang S et al. Chloroplast RNA-binding protein RBD1 promotes chilling tolerance through 23S rRNA processing in *Arabidopsis*. *PLoS Genet*. 2016;12:e1006027. 27138552 10.1371/journal.pgen.1006027 PMC4854396

[54] Asakura Y, Barkan A. *Arabidopsis* orthologs of maize chloroplast splicing factors promote splicing of orthologous and species-specific group II introns. *Plant Physiol*. 2006;142:1656–63. 17071648 10.1104/pp.106.088096 PMC1676066

[55] Tanabe N, Yoshimura K, Kimura A et al. Differential expression of alternatively spliced mRNAs of *Arabidopsis* SR protein homologs, atSR30 and atSR45a, in response to environmental stress. *Plant Cell Physiol*. 2007;48:1036–49. 17556373 10.1093/pcp/pcm069

[56] Soltis DE, Smith SA, Cellinese N et al. Angiosperm phylogeny: 17 genes, 640 taxa. *Am J Bot*. 2011;98:704–30. 21613169 10.3732/ajb.1000404

[57] Song X-M, Wang JP, Sun PC et al. Preferential gene retention increases the robustness of cold regulation in Brassicaceae and other plants after polyploidization. *Hortic Res*. 2020;7:20. 32133148 10.1038/s41438-020-0253-0 PMC7035258

[58] Peterson-Burch BD, Nettleton D, Voytas DF. Genomic neighborhoods for *Arabidopsis* retrotransposons: a role for targeted integration in the distribution of the Metaviridae. *Genome Biol*. 2004;5:R78. 15461796 10.1186/gb-2004-5-10-r78 PMC545598

[59] Jin W, Melo JR, Nagaki K et al. Maize centromeres: organization and functional adaptation in the genetic background of oat. *Plant Cell*. 2004;16:571–81. 14973167 10.1105/tpc.018937 PMC385273

[60] Miller WJ, Capy P. Applying mobile genetic elements for genome analysis and evolution. *Mol Biotechnol*. 2006;33:161–74. 16757803 10.1385/MB:33:2:161

[61] Todorovska E . Retrotransposons and their role in plant—genome evolution. *Biotechnol Biotechnol Equip*. 2014;21:294–305.

[62] Beulé T, Agbessi MDT, Dussert S et al. Genome-wide analysis of LTR-retrotransposons in oil palm. *BMC Genomics*. 2015;16:795. 26470789 10.1186/s12864-015-2023-1 PMC4608283

[63] Koren S, Walenz BP, Berlin K et al. Canu: scalable and accurate long-read assembly via adaptive k-mer weighting and repeat separation. *Genome Res*. 2017;27:722–36. 28298431 10.1101/gr.215087.116 PMC5411767

[64] Vaser R, Sović I, Nagarajan N et al. Fast and accurate de novo genome assembly from long uncorrected reads. *Genome Res*. 2017;27:737–46. 28100585 10.1101/gr.214270.116 PMC5411768

[65] Walker BJ, Abeel T, Shea T et al. Pilon: an integrated tool for comprehensive microbial variant detection and genome assembly improvement. *PLoS One*. 2014;9:e112963. 25409509 10.1371/journal.pone.0112963 PMC4237348

[66] Li H, Durbin R. Fast and accurate short read alignment with Burrows-Wheeler transform. *Bioinformatics*. 2009;25:1754–60. 19451168 10.1093/bioinformatics/btp324 PMC2705234

[67] Burton JN, Adey A, Patwardhan RP et al. Chromosome-scale scaffolding of de novo genome assemblies based on chromatin interactions. *Nat Biotechnol*. 2013;31:1119–25. 24185095 10.1038/nbt.2727 PMC4117202

[68] Marchler-Bauer A, Lu S, Anderson JB et al. CDD: a conserved domain database for the functional annotation of proteins. *Nucleic Acids Res*. 2011;39:D225–9. 21109532 10.1093/nar/gkq1189 PMC3013737

[69] Koonin EV, Fedorova ND, Jackson JD et al. A comprehensive evolutionary classification of proteins encoded in complete eukaryotic genomes. *Genome Biol*. 2004;5:R7. 14759257 10.1186/gb-2004-5-2-r7 PMC395751

[70] Ogata H, Goto S, Sato K et al. KEGG: Kyoto Encyclopedia of Genes and Genomes. *Nucleic Acids Res*. 1999;27:29–34. 9847135 10.1093/nar/27.1.29 PMC148090

[71] Boeckmann B, Bairoch A, Apweiler R et al. The SWISS-PROT protein knowledgebase and its supplement TrEMBL in 2003. *Nucleic Acids Res*. 2003;31:365–70. 12520024 10.1093/nar/gkg095 PMC165542

[72] Altschul SF, Gish W, Miller W et al. Basic local alignment search tool. *J Mol Biol*. 1990;215:403–10. 2231712 10.1016/S0022-2836(05)80360-2

[73] Dimmer EC, Huntley RP, Alam-Faruque Y et al. The UniProt-GO annotation database in 2011. *Nucleic Acids Res*. 2012;40:D565–70. 22123736 10.1093/nar/gkr1048 PMC3245010

[4] Harris MA, Clark J, Ireland A et al. The Gene Ontology (GO) database and informatics resource. *Nucleic Acids Res*. 2004;32:D258–61. 14681407 10.1093/nar/gkh036 PMC308770

[75] Conesa A, Götz S, García-Gómez JM et al. Blast2GO: a universal tool for annotation, visualization and analysis in functional genomics research. *Bioinformatics*. 2005;21:3674–6. 16081474 10.1093/bioinformatics/bti610

[76] Zheng Y, Jiao C, Sun H et al. iTAK: a program for genome-wide prediction and classification of plant transcription factors, transcriptional regulators, and protein kinases. *Mol Plant*. 2016;9:1667–70. 27717919 10.1016/j.molp.2016.09.014

[77] Letunic I, Bork P. 20 years of the SMART protein domain annotation resource. *Nucleic Acids Res*. 2018;46:D493–d496. 29040681 10.1093/nar/gkx922 PMC5753352

[78] Prakash A, Jeffryes M, Bateman A et al. The HMMER web server for protein sequence similarity search. *Curr Protoc Bioinformatics*. 2017;60:3.15.11–13.15.23. 10.1002/cpbi.40 29220076

[79] Finn RD, Bateman A, Clements J et al. Pfam: the protein families database. *Nucleic Acids Res*. 2014;42:D222–30. 24288371 10.1093/nar/gkt1223 PMC3965110

[80] Tarailo-Graovac M, Chen N. Using RepeatMasker to identify repetitive elements in genomic sequences. *Curr Protoc Bioinformatics*. 2009; Chapter 4:Unit 4.10. 10.1002/0471250953.bi0410s25 19274634

[81] Claire H, Arnoux S, Moisset M et al. PASTEC: an automatic transposable element classification tool. *PLoS One*. 2014;9:e91929. 24786468 10.1371/journal.pone.0091929 PMC4008368

[82] Xu Z, Wang H. LTR_FINDER: an efficient tool for the prediction of full-length LTR retrotransposons. *Nucleic Acids Res*. 2007;35:W265–8. 17485477 10.1093/nar/gkm286 PMC1933203

[83] Ou S, Jiang N. LTR_retriever: a highly accurate and sensitive program for identification of long terminal repeat retrotransposons. *Plant Physiol*. 2018;176:1410–22. 29233850 10.1104/pp.17.01310 PMC5813529

[84] Katoh K, Standley DM. MAFFT multiple sequence alignment software version 7: improvements in performance and usability. *Mol Biol Evol*. 2013;30:772–80. 23329690 10.1093/molbev/mst010 PMC3603318

[85] Price MN, Dehal PS, Arkin AP. FastTree: computing large minimum evolution trees with profiles instead of a distance matrix. *Mol Biol Evol*. 2009;26:1641–50. 19377059 10.1093/molbev/msp077 PMC2693737

[86] Emms DM, Kelly S. OrthoFinder: phylogenetic orthology inference for comparative genomics. *Genome Biol*. 2019;20:238. 31727128 10.1186/s13059-019-1832-y PMC6857279

[87] Han MV, Thomas GW, Lugo-Martinez J et al. Estimating gene gain and loss rates in the presence of error in genome assembly and annotation using CAFE 3. *Mol Biol Evol*. 2013;30:1987–97. 23709260 10.1093/molbev/mst100

[88] Sanderson MJ . r8s: inferring absolute rates of molecular evolution and divergence times in the absence of a molecular clock. *Bioinformatics*. 2003;19:301–2. 12538260 10.1093/bioinformatics/19.2.301

[89] Tang H, Bowers JE, Wang X et al. Synteny and collinearity in plant genomes. *Science*. 2008;320:486–8. 18436778 10.1126/science.1153917

[90] Chen S, Zhou Y, Chen Y et al. Fastp: an ultra-fast all-in-one FASTQ preprocessor. *Bioinformatics*. 2018;34:i884–90. 30423086 10.1093/bioinformatics/bty560 PMC6129281

[91] Kim D, Langmead B, Salzberg SL. HISAT: a fast spliced aligner with low memory requirements. *Nat Methods*. 2015;12:357–60. 25751142 10.1038/nmeth.3317 PMC4655817

[92] Trapnell C, Roberts A, Goff L et al. Differential gene and transcript expression analysis of RNA-seq experiments with TopHat and cufflinks. *Nat Protoc*. 2012;7:562–78. 22383036 10.1038/nprot.2012.016 PMC3334321

[93] McKenna A, Hanna M, Banks E et al. The Genome Analysis Toolkit: a MapReduce framework for analyzing next-generation DNA sequencing data. *Genome Res*. 2010;20:1297–303. 20644199 10.1101/gr.107524.110 PMC2928508

[94] Wang K, Li M, Hakonarson H. ANNOVAR: functional annotation of genetic variants from high-throughput sequencing data. *Nucleic Acids Res*. 2010;38:e164. 20601685 10.1093/nar/gkq603 PMC2938201

[95] Li H . Minimap2: pairwise alignment for nucleotide sequences. *Bioinformatics*. 2018;34:3094–100. 29750242 10.1093/bioinformatics/bty191 PMC6137996

[96] Anders S, Huber W. Differential expression analysis for sequence count data. *Genome Biol*. 2010;11:R106. 20979621 10.1186/gb-2010-11-10-r106 PMC3218662

[97] Ernst J, Bar-Joseph Z. STEM: a tool for the analysis of short time series gene expression data. *BMC Bioinformatics*. 2006;7:191. 16597342 10.1186/1471-2105-7-191 PMC1456994

[98] Trincado JL, Entizne JC, Hysenaj G et al. SUPPA2: fast, accurate, and uncertainty-aware differential splicing analysis across multiple conditions. *Genome Biol*. 2018;19:40. 29571299 10.1186/s13059-018-1417-1 PMC5866513

