# Supplementary material for: The reference genome and full-length transcriptome of pakchoi provide insights into cuticle formation and heat adaption
Source: Hortic Res. 2022 May 26;9:uhac123. doi: 10.1093/hr/uhac123 (PMC9358696; doi:10.1093/hr/uhac123)
Supplement: Suppl_uhac123 [file suppl_uhac123.zip › supplemental files.docx]

**Supplemental file -The reference genome and full-length transcriptome of pakchoi provide insights into the cuticle formation and heat adaption**

### Supplementary Figures

**Fig. S1.** The *k-mer* distribution of sequencing reads from the pakchoi genome.

**Fig. S2.** The Hi-C chromatin interaction map for the 10 chromosomes of genome.

**Fig. S3.** Analysis of gene families and phylogenetic and whole-genome duplication.

**Fig. S4.** GO function enrichment of expansion genes in pakchoi.

**Fig. S5.** Prediction of conserved domain for expansion genes in pakchoi.

**Fig. S6.** The distribution of F-box genes among 9 representative *B. rapa* subspecies and *B. oleracea* (BOL), and *A. thaliana* (ATH).

**Fig. S7.** The analyses of TE comparison.

**Fig. S8.** The overview of cuticle metabolism pathways.

**Fig. S9.** Evolutionary scenario of Chinese cabbage (BRP) and pakchoi (BRC) from an ancestral 8-chromosome karyotype of Brassicaceae and subsequent Brassiceae genome triplication.

**Fig. S10.** Phenotypic characterization of glaucous varieties and glossy varieties.

**Fig. S11.** Identification of the hot-region for cuticle phenotype through association analysis.

**Fig. S12.** The conserved domains of protein encoded by *BrcCER1* gene.

**Fig. S13.** The comparison of 17 glaucous and glossy cultivars.

**Fig. S14.** The position of T-DNA insertion on *CER1* in *cer1-2* mutant.

**Fig. S15.** Time series changes in plants treated in high temperature experiments.

**Fig. S16.** Dynamic progression of high temperature experiments transcriptome in PC-fu (tolerant to high-temperature varieties) and JP20 (sensitive to high-temperature varieties).

**Fig. S17.** The expression pattern of genes involved in chlorophyll biosynthesis.

**Fig. S18.** DAS genes and DTU showed dynamic expression trend under time series high temperature treatment.

**Fig. S19.** Distribution of different types of alternative splicing events in PC-fu and PC20.


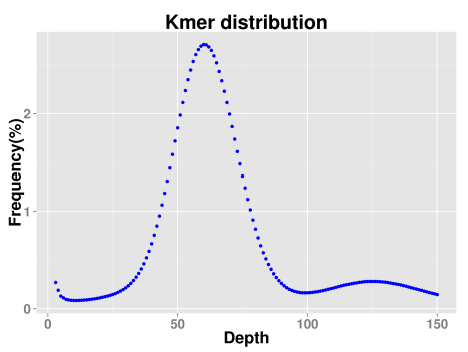


2

1

Frequency (%)

0

150

100

50

0

Depth

**Fig. S1: The *k-mer* distribution of sequencing reads from the pakchoi genome.** The genome size, content of repeat sequence, heterozygosity and GC content were calculated based on depth.


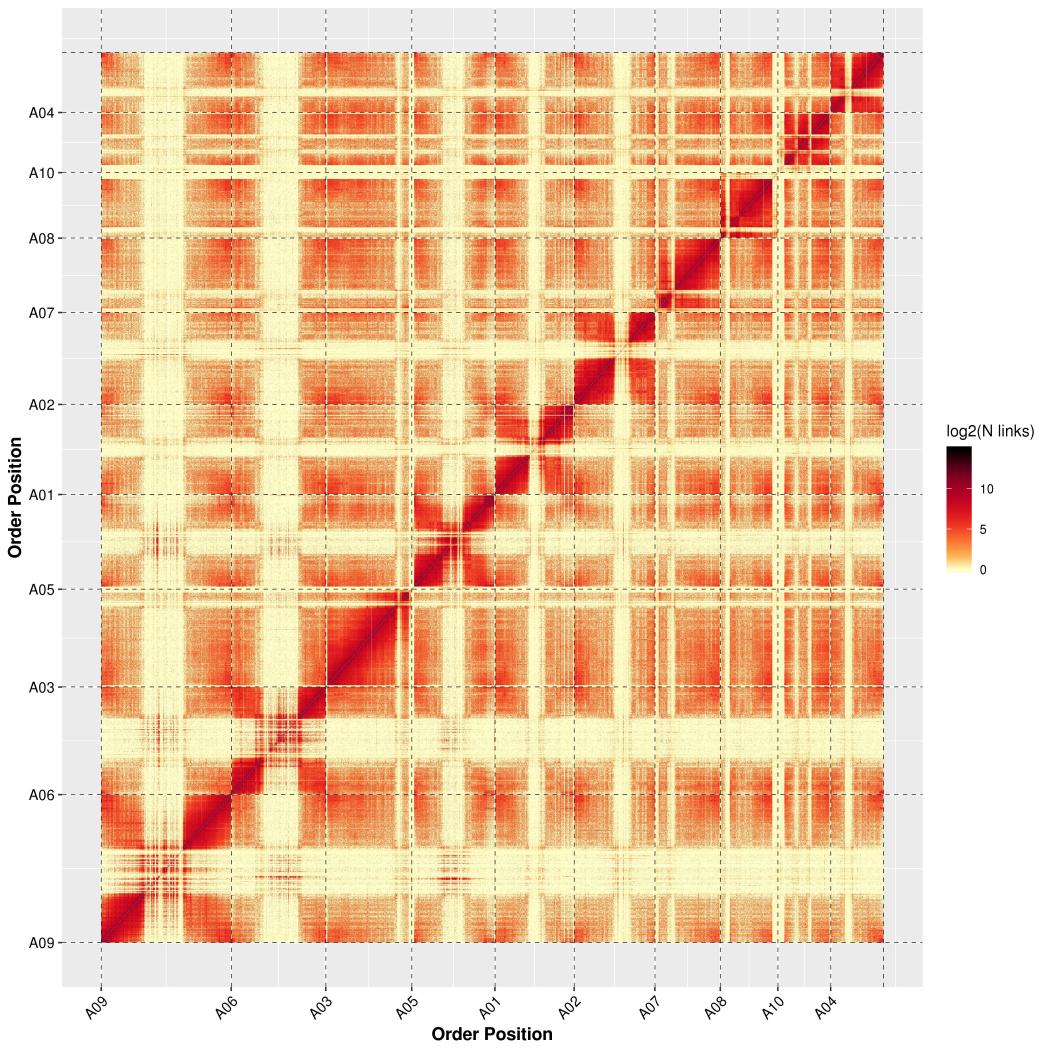


Order Position

Order Position

A09

A06

A03

A05

A01

A02

A07

A08

A04

A10

A09

A06

A03

A02

A05

A01

A07

A08

A04

A10

log2(N links)

15.0

10.0

5.0

0.0

**Fig. S2. The Hi-C chromatin interaction map for the 1-10 chromosomes of pakchoi genome.**

B

A


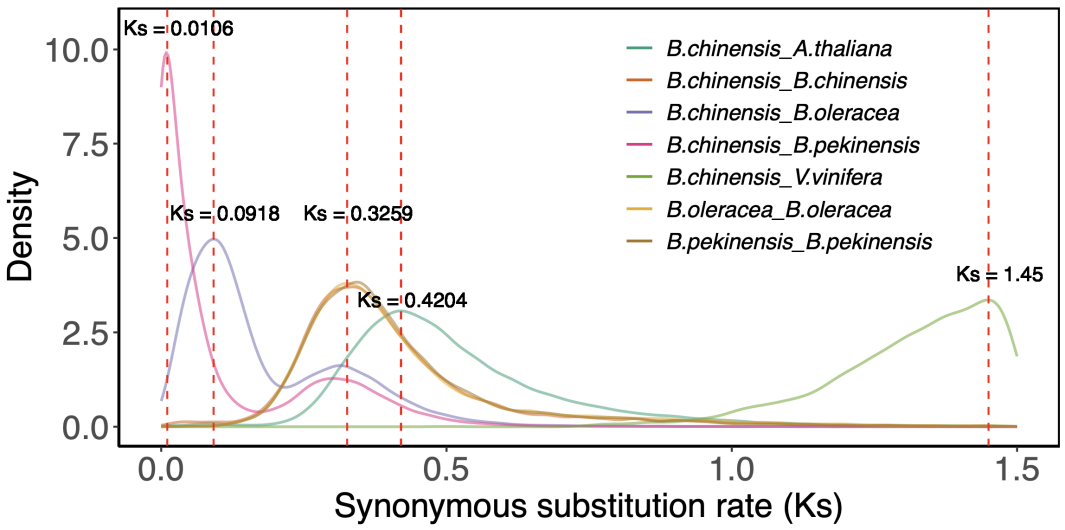


**Fig. S3. Analysis of gene families and phylogenetic and whole-genome duplication.** **A.** Expansion and contraction of gene families among the 10 plant species. Phylogenetic tree was constructed based on 533 high-quality 1:1 single-copy orthologous genes. Pie diagram on each branch of the tree represents the proportion of genes undergoing gain (red) or loss (green) events. Number at root (24,977) denotes the total number of gene families predicted in the most recent common ancestor (MRCA). The numerical value beside each node shows the estimated divergent time of each node (MYA). **B.** Genome duplication in dicot genomes (BRC, BRP, *B. oleracea*, and *A. thaliana*) revealed by synonymous substitution rate (Ks) analyses.

**Fig. S4. GO function enrichment of expansion genes in pakchoi**.

**Fig. S5. Prediction of conserved domain for expansion genes in pakchoi.**

**Fig. S6. The Distribution of F-box genes among 9 representative *B. rapa* subspecies and *B. oleracea* (BOL), and *A. thaliana* (ATH).**

**A**

Group

BRC

BRP

B

**Fig. S7. The analyses of TE comparison. A.** Phylogeny of the *Gypsy-like* elements as an example of LTR-RTs of the syntenic regions in pakchoi (BRC) and Chinese cabbage (BRK). The neighbor-joining (NJ) trees were generated based on the conserved RT domain nucleotide sequences using MEGA X. **B.** TE total length of syntenic regions in BRC and other *B. rapa* subspecies. Retro, Retro transposon. DNA, DNA transposon. Blue columns represent pakchoi and red columns represent other subspecies.


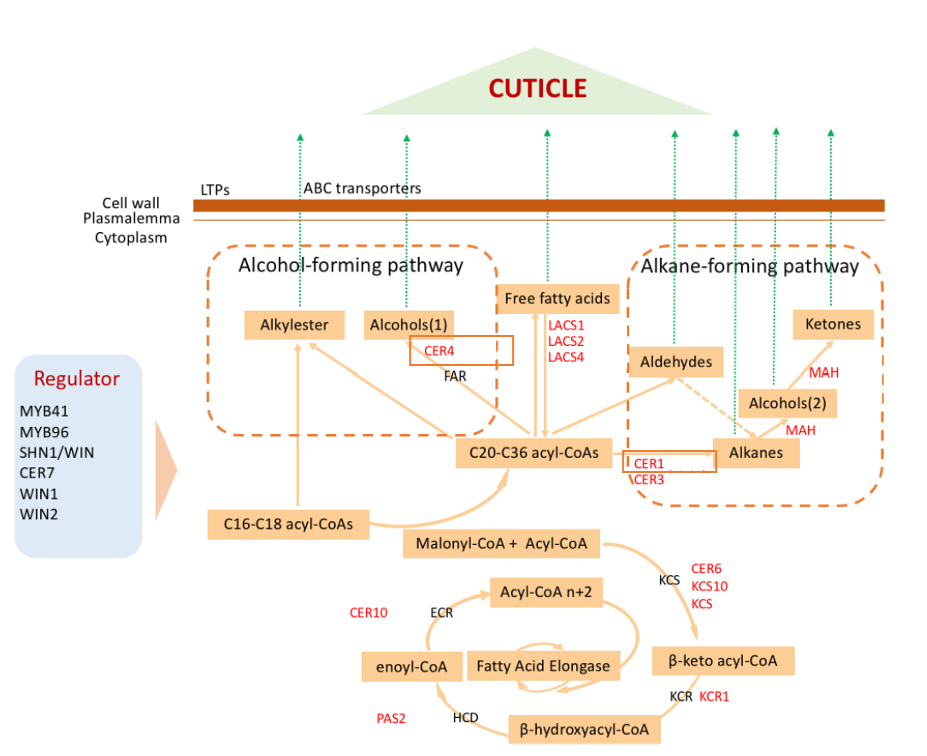


**Fig. S8. The overview of cuticle metabolism pathways.**

Brassicaceae ansestor

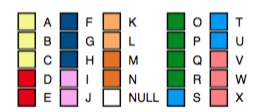


BRC *n=10*

**Fig. S9. Evolutionary scenario of pakchoi (BRC) from an ancestral 8-chromosome karyotype of Brassicaceae and subsequent Brassiceae genome triplication.** The 24 ancestral blocks (labelled A-X) were defined and coloured in ancestor of Brassicaceae as described previously. The distribution of ancestral block in BRC obtained by alignment analysis between *A. thaliana* and BRC.


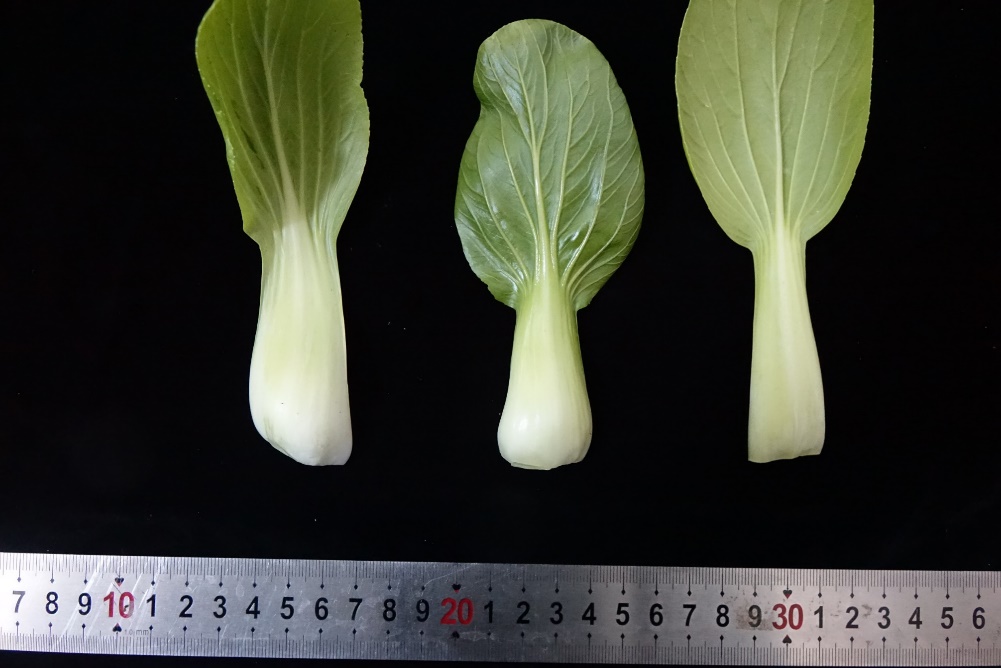

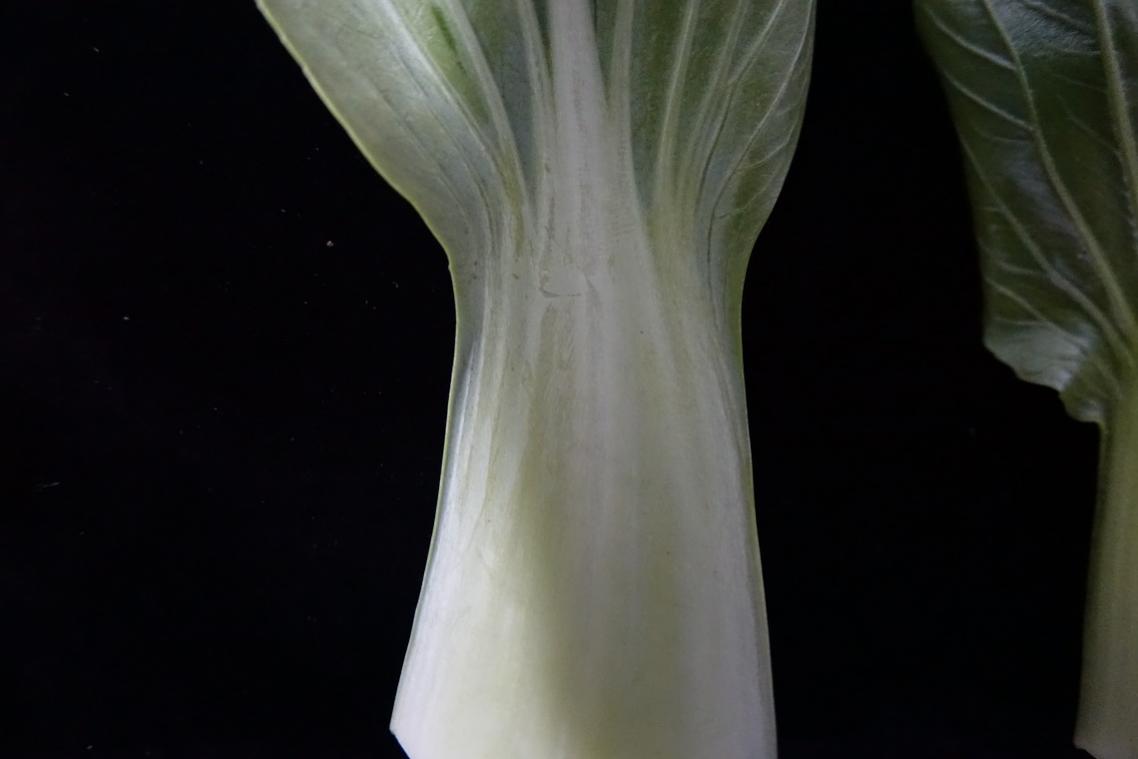

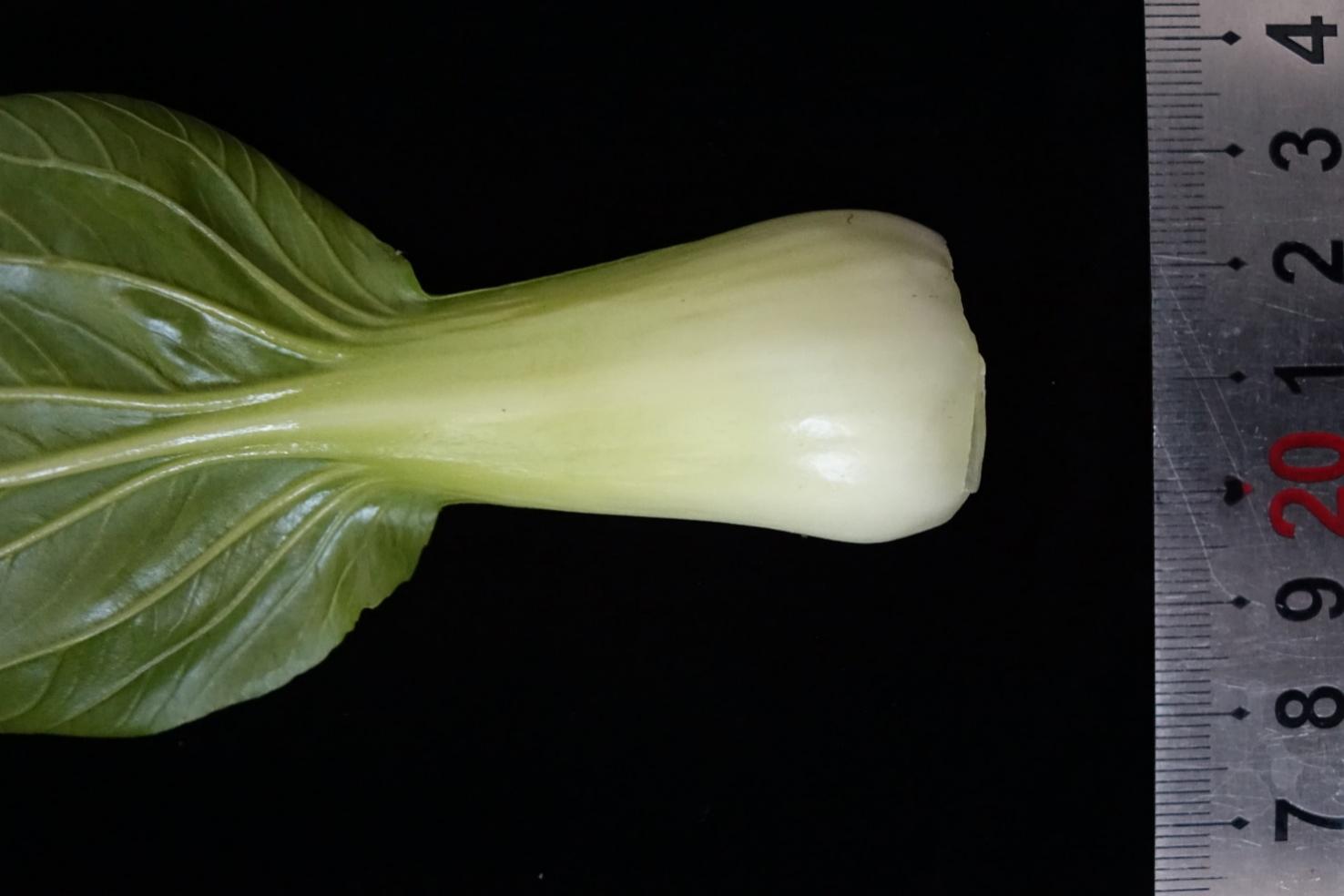


**B**

**C**











Glossy varieties

Glaucous varieties

**Leaf**

**Petiole**

Glossy varieties

Glaucous varieties

Glaucous varieties

Glossy varieties

**A**

Glaucous varieties

Glossy varieties

D

**Fig. S10. Phenotypic characterization of glaucous varieties and glossy varieties. A.** Cuticular wax phenotype on the leaf and petiole of two varieties. **B** and **C.** Close-up view of the squared areas in A. **D.** SEM analysis of the wax crystals on the leaf surface and petiole of two pakchoi varieties at the rosette leaves stage. Scale bar in (A-C), 2 cm; (D) 50 μm.


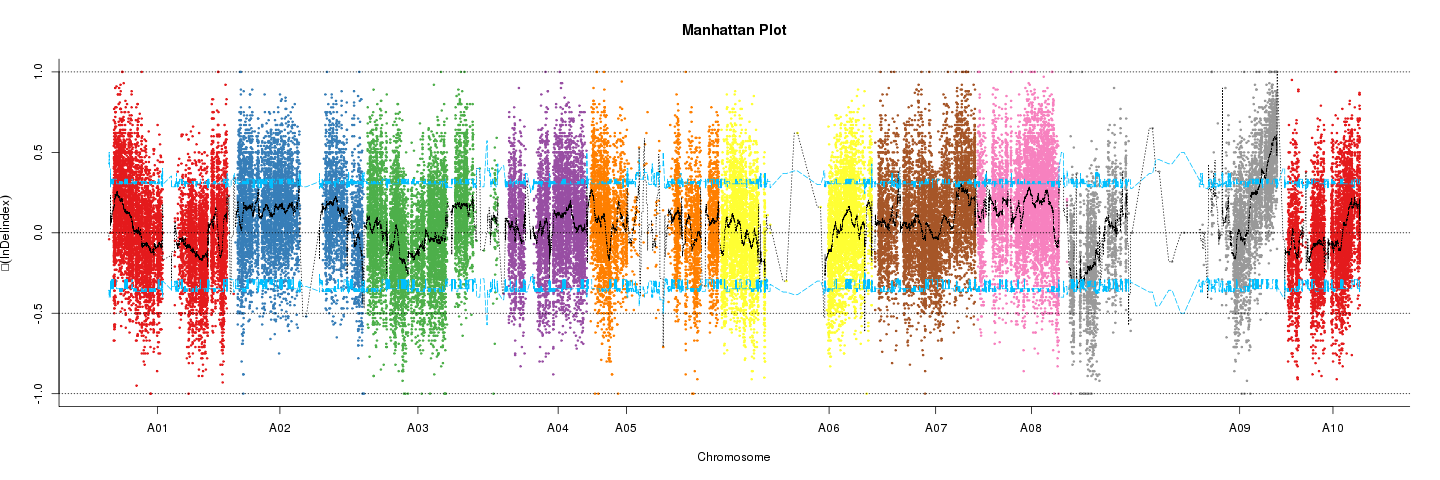

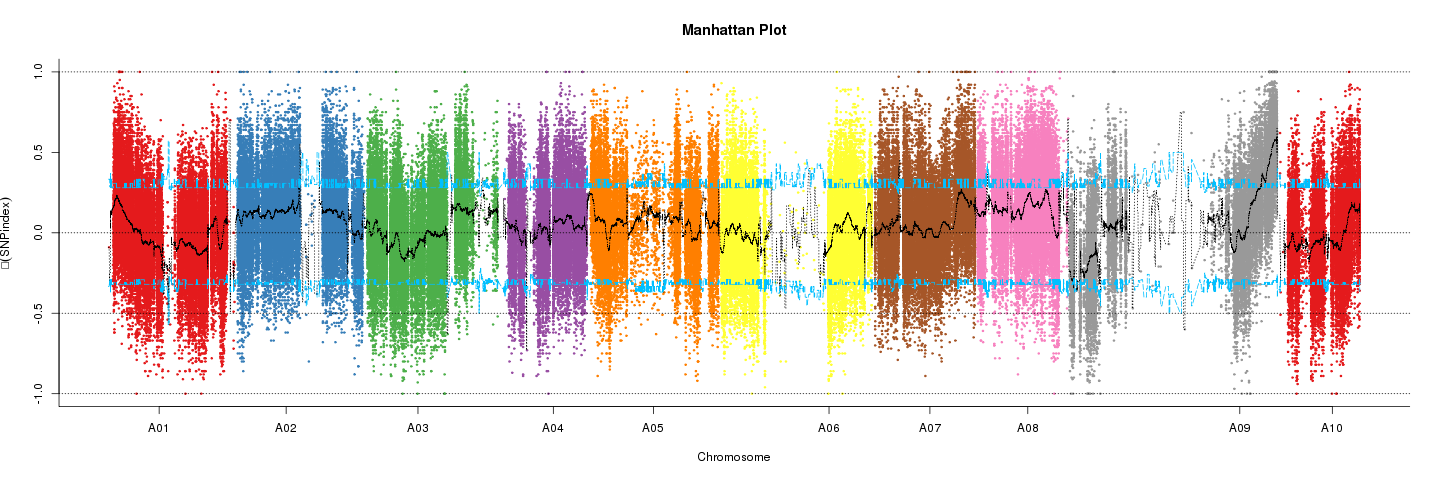


A

B

**Δ SNP index**

**Δ Indel index**

**Chromosome**

**A01**

**A02**

**A03**

**A04**

**A05**

**A06**

**A07**

**A08**

**A09**

**A10**

**Chromosome**

**A01**

**A02**

**A03**

**A04**

**A05**

**A06**

**A07**

**A08**

**A09**

**A10**

**-1.0**

**-0.5**

**0.0**

**0.5**

**1.0**

**-1.0**

**-0.5**

**0.0**

**0.5**

**1.0**

**Fig. S11. Identification of the hot-region for cuticle phenotype through association analysis.** X-axis represents the position of 12 chromosomes of pakchoi and Y-axis represents the Δ (SNP-index) or Δ (indel-index). The color dots represent Δ (SNP-index) or Δ (indel-index) value of every locus. The black lines value of fitting results. A: The Δ (SNP-index). B: The Δ (indel-index).


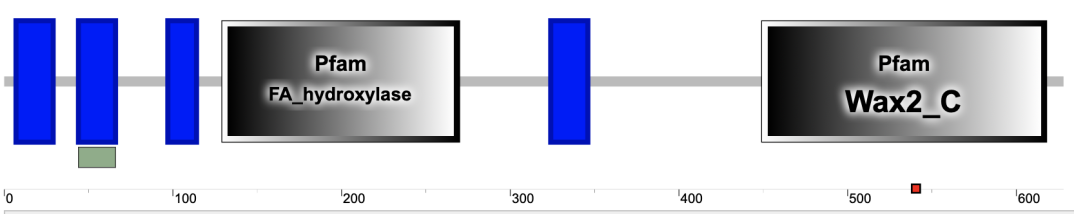


**Fig. S12. The conserved domains of protein encoded by *BrcCER1* gene.** The red boxes represent premature termination of transcription due to loss of bases in glossy varieties, resulting in loss of function.


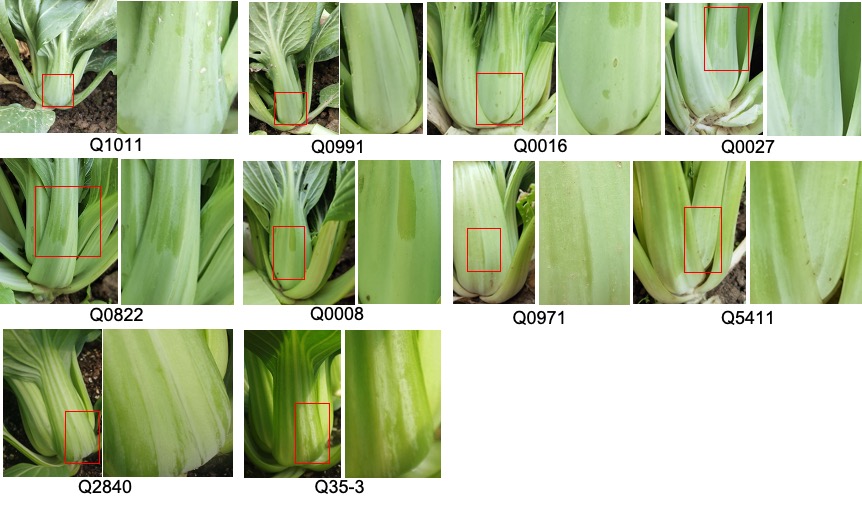


B

A

**
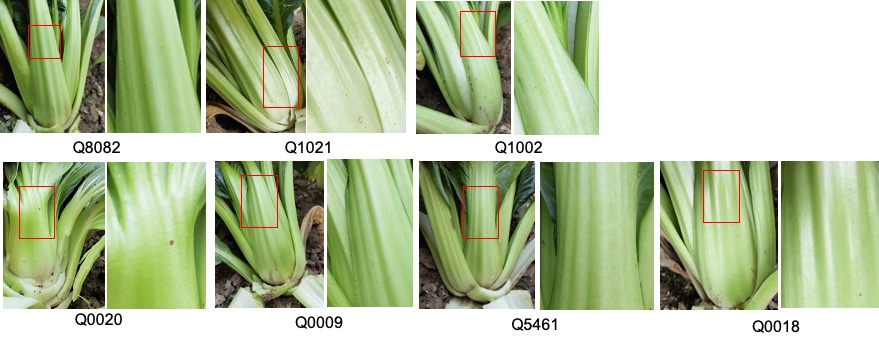
**

C

**Figure S13. The comparison of 17 glaucous and glossy cultivars.** **A.** Phenotype of 10 glaucous cultivars. **B.** Phenotype of 7 glossy cultivars. **C.** The comparison of flanking nucleic acid sequences of the mutation site of *BrcCER1* in those cultivars.

**ATG**

***cer1-2***

**Salk_014839**

**-Coding region**

**-Untranslated region**

**T-DNA insert (-266 bp)**

**LP1**

**RP1**

**Fig. S14. The position of T-DNA insertion on *CER1* in *cer1-2* mutant.**


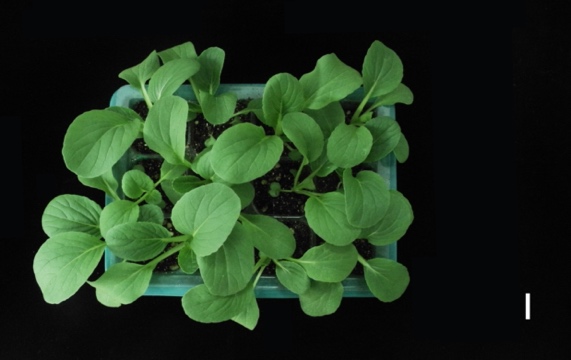

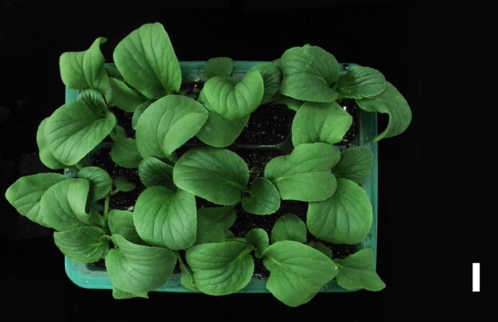

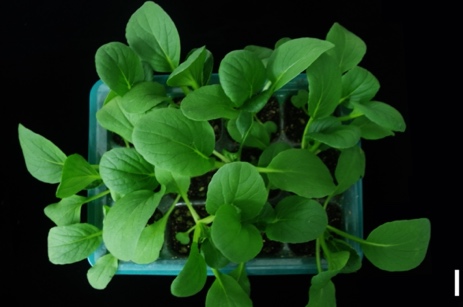

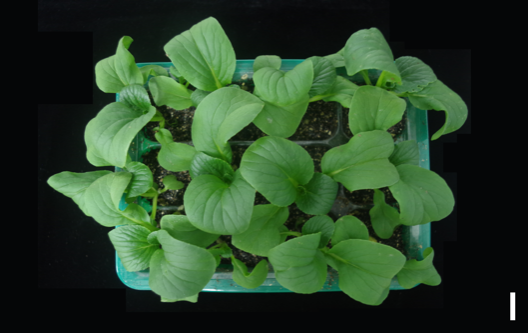

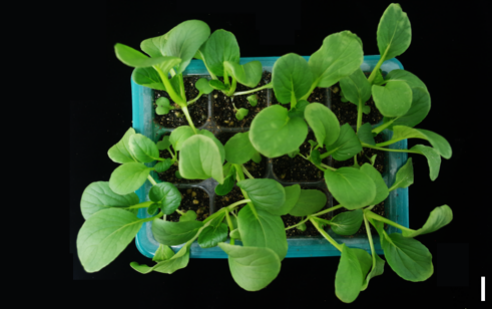

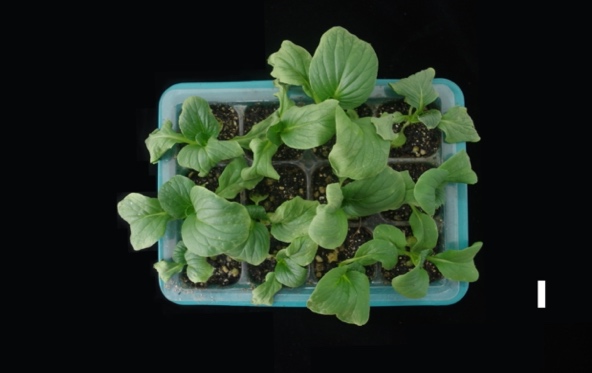


**Tolerant**

**Sensitive**

**0 h**

**12 h**

**24 h**


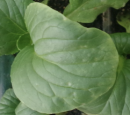

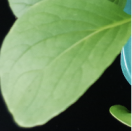


**Fig. S15. Time series changes in plants treated in high temperature experiments.** The leaves of PC-fu (tolerant to high-temperature varieties) and JP20 (sensitive to high-temperature varieties) were harvest and pooled for each sampling time (0 h, 12 h and 24 h under 40°C high temperature treatment).

**Fig. S16. Dynamic progression of high temperature experiments transcriptome in PC-fu (tolerant to high-temperature varieties) and JP20 (sensitive to high-temperature varieties).** Ten major gene expression patterns (C1-C10) were respectively performed along three time point in PC-fu and JP20. The right side of the plot is functional category enrichment among the ten major clusters.

Log_10_(CPM)

0h

12h

24h

0h

12h

24h

Tolerance

Sensitivity

**Fig. S17. The expression pattern of genes involved in chlorophyll biosynthesis in PC-fu (tolerant to high-temperature varieties) and JP20 (sensitive to high-temperature varieties) under time series high temperature treatment.**

0h

12h

24h

0h

12h

24h

Tolerance

Sensitivity

0h

12h

24h

0h

12h

24h

Tolerance

Sensitivity

Z-score

2

1

0

-1

-2

Z-score

A

B

2

1

0

-1

-2

**Fig. S18. DAS genes and DTU showed dynamic expression trend in PC-fu (tolerant to high-temperature varieties) and JP20 (sensitive to high-temperature varieties) under time series high temperature treatment.**

**Fig. S19. Distribution of different types of alternative splicing events in PC-fu and JP20.**

### Supplemental experimental procedures

**Section S1-** Sample preparation, library construction, genome sequencing

**Section S2-** genome assembly and validation

**Section S3-** Gene prediction and functional annotation

**Section S4-** Gene clusters and duplication

**Section S5-** The expression bias analysis of three subgenome homologs

**Section S6-** RNA-seq analysis

**Section S7-** BSA-seq experiment

**Section S8-** Full-length transcriptome sequencing and analysis

**References**

### Supplemental experimental procedures

### Section S1-Sample preparation, library construction and genome sequencing

**1. 1 Sample preparation**

For the genome sequencing, cultivar PC-fu which is widely used as a parent in breeding with rich cuticle and high temperature-resistant were selected and planted in soil at the Biotron at Fujian Agricultural and Forestry University in December 2018 under the following conditions: 22 °C (16 h day/8 h night) and 75% relative humidity. An individual leaves were harvested and immediately frozen in liquid nitrogen after collection, followed by preservation at -80°C in the laboratory prior to DNA extraction. High-quality genomic DNA was extracted from leaves using CTAB method^1^. The leaves, stems, capsules, roots, and flowers were collected for RNA extraction via the CTAB-LiCl method.

### 1.2 Estimation of genome size using *k-mer* analysis

The qualified genomic DNA was randomly disrupted by ultrasonic oscillation to generate the fragments of 350 bp, and then a small fragment sequencing library was constructed by terminal repair, the addition of A bases and linkers, target fragment selection and PCR. The library was subjected to paired-end 150 bp (PE 150) sequencing using the Illumina platform. The data were subjected to quality control and used for analysis. The results showed that a total of 36.70 Gb of data were obtained (**Table S1**). The data were subjected to quality control and used for analysis.

A *k-mer* is an oligonucleotide sequence of length k extracted from the sliding windows of sequencing data. Under the premise of a uniform distribution of sequencing reads, the following formula is obtained:

$$Genomic size=\frac{total number of bases}{average sequencing depth}$$

$$=\frac{total kmer}{median kmer depth}$$

A *k-mer* map of k=19 was constructed using the 350-bp library data (**Fig. S1**) for the evaluation of genome size, the repeat sequence ratio, and heterozygosity. The main peak corresponding to the *k-mer* depth was 58, which was the average *k-mer* depth. A sequence in which the *k-mer* depth appeared to be more than twice the depth of the main peak (depth value, 117) was considered a repeat sequence. A *k-mer* depth was half of the main peak (depth value, 29), indicating that the sequence was heterozygous. The total number of *k-mer*s obtained from the sequencing data was 32,278,151,809. After the removal of *k-mer*s with an abnormal depth, a total of 29,335,932,217 *k-mer*s were used for genome size estimation, and the calculated genome length was ∼501.04 Mb. According to the *k-mer* distribution, the estimated repeat sequence ratio was ∼63.07%. There was no obvious heterozygous peak, and the heterozygosity was low, at 0.09%.

### 1.3 library construction and genome sequencing

Genomic DNA was extracted and sequenced following the instructions of the Ligation Sequencing Kit (Nanopore, Oxfordshire, UK). The isolated DNA were checked using 0.35% agarose gel electrophoresis and a Qubit fluorimeter (Thermo Fisher, CA, USA), respectively. This high-quality DNA was used for subsequent Nanopore. To generate Oxford Nanopore long reads, approximately 15 μg of genomic DNA was size-selected (30–80 kb) with a BluePippin (Sage Science, Beverly, MA, USA), and processed according to the Ligation Sequencing Kit 1D (SQK-LSK109) protocol. Briefly, DNA fragments were repaired using the NEBNext FFPE Repair Mix (New England Biolabs). After end-reparation and 3’-adenylation with the NEBNext End repair/dA-tailing Module reagents (New England Biolabs), the Oxford Nanopore sequencing adapters were ligated using NEBNext Quick Ligation Module (E6056) (New England Biolabs). The final library was sequenced on the Nanopore PromethION platform using 2low cells, according to the manufacturer’s protocols (PromethION, RRID:SCR_017987). Approximately 62.71 Gb of data were obtained. After data quality control, the final data volume was 60.69 Gb (**Table S1**). The Guppy pipeline of MinKNOW software was used to conduct base calling of raw signal data and convert the fast5 files into fastq files. These raw data were then filtered to remove short reads (<5 kb) and the reads with low-quality bases and adapter sequences.

A Hi-C sample library was constructed from genomic DNA from the fresh leaves. The main procedures included cross-linking the DNA, restriction enzyme digestion, end repair, DNA cyclization, and DNA purification. The library was sequenced on the Illumina HiSeq 4000 platform. A total of 53.44 Gb of clean data were obtained, and the Q30 was 93.04%.

### Section S2-genome assembly and validation

### 2.1 Genome assembly based on Nanopore and Hi-C data

Canu software^2^ was conducted for initial read correction, and then the assembly of the corrected reads were executed by WTDBG2 (<https://github.com/ruanjue/wtdbg>) and smartdenovo software (<https://github.com/ruanjue/smartdenovo>) respectively. Quickmerge software^3^ was used to integrate WTDBG2 and smartdenovo assembly results, and then Racon software was used to correct consensus assembly. Finally, the Illumina reads data were polished by Pilon software^4^ with default settings to obtain the final version of genome.

For Hi-C sequencing, the fresh leaves of pakchoi were used for library preparation^5^. HiC-Pro (v2.8.1)^6^ was performed to find the valid reads from unique mapped read pairs. Clean Hi-C data were aligned to the primary draft assembly using BWA-MEM (v0.7.10-r789) ^7^. Ultimately, 1,364 scaffolds were anchored to 10 pseudo-molecules using LACHESIS based on Hi-C data ^8^.

### 2.2 Evaluation of assembly quality

To evaluate the completeness and accuracy of genome assembly, the Illumina reads were aligned to the pakchoi assembly using BWA-MEM^7^. CEGMA^9^ was further performed to detect the core eukaryotic genes (CEGs) in the genome and BUSCO analysis^10^ was finally performed to assess completeness of the genome assembly.

### Supplementary section S3- Gene prediction and functional annotation

### 3.1 ab initio gene prediction and homology-based gene prediction

The protein-coding genes of the pakchoi genome were predicted using a combination of *ab initio* prediction, homolog-based prediction and RNA-seq based prediction. Genscan^11^, Augustus (v2.4)^12^, GlimmerHMM (v3.0.4)^13^, GeneID (v173 1.4)^14^, and SNAP (v2006-07-28)^15^ were used for ab initio-based gene prediction in pakchoi genome assembly. We aligned the protein sequences from *Arabidopsis thaliana* (*A. thaliana*), *Brassica napus* (*B. napus*) and *B. rapa* ssp*. pekinensis* (BRP) to the pakchoi genome using GeMoMa^16,17^. For the RNA-seq-based method, we mapped the transcripts generated from Illumina short-read sequencing technologies to the genome assembly using HISAT2 (v2.0.4)^18^, the retained high-quality clean reads were first assembled by Stringtie (v1.2.3)^19^, and then the gene prediction was performed using TransDecoder (http://transdecoder.github.io) (v2.0), GeneMarkS-T (v5.1)^20^, and PASA (v2.0.2)^21^. Finally, we combined all evidence from the above 3 methods into the consensus gene sets using the EVidenceModeler pipeline^22^.

### 3.2 Gene function annotation

To annotate the function of the protein-coding genes, they were aligned to the NCBI non-redundant protein sequences (NR)^23^, eukaryotic orthologous groups of proteins (KOG)^24^, Kyoto Encyclopedia of Genes and Genomes (KEGG)^25^ and TrEMBL^26^ databases using BLAST (v2.2.31)^27^ with an E-value cutoff of 1E-5. Gene ontology (GO) ^28,29^ annotation was performed with Blast2GO (v4.1)^30^. For non-coding RNA prediction, we annotated the transfer RNAs (tRNAs) genes in the pakhoi genome using tRNAscan-SE (v1.3.1)^31^. Furthermore, Blastn^27^ was performed to search for ribosomal RNAs (rRNAs) and microRNAs based on Rfam (v13.0)^32^ database. We also annotated genes encoding transcription factors using iTAK programmer^33^. The putative domains of proteins were identified using SMART^34^ and HMMER^35^ based on PFAM database^36^.

### 3.3 Repeat annotation

RepeatModeler^37^ was used to develop a *de novo* repeat library, which used two *de novo* repeat-finding programmes (RECON and RepeatScout) for identification of the repeat families. The database was classified by PASTEClassifier^38^, and then merged with the library of RepBase to make the final repetitive sequence database. RepeatMasker was employed to estimate repeat copies, proportion and distribution into the genome^39^.

Intact, long terminal repeat retrotransposons (LTR-RTs) were identified from both pakchoi and Chinese cabbage genome assemblies using LTR_harvest (-motif TGCA -motifmis 1)^40^ and LTR_Finder (-D 15000 -d 1000 -L 7000 -l 100)^41^. The resulting outputs (.scn) were fed into the LTR_retriever programmer^42^ to integrate two above results. Copy number, distribution and divergence time of LTR-RTs were comparatively analyzed between the two reference genomes. Phylogenetic analysis was constructed using the LTR-RTs sequences of *copia* and *gyspy* from the two *B. rapa* subspecies genomes. LTR-RTs sequences of *copia* and *gyspy* were aligned separately by the MAFFT aligner^43^, and a tree was generated using FastTree^44^ and visualized by Ggtree^45^.

### Section S4- Gene clusters and duplication

### 4.1 Gene families and phylogenetic analysis

OrthoFinder package (v2.2.7)^46^ was used to identify gene families/clusters between the pakchoi and 8 other plant species, including monocots (*Oryza sativa* and *Sorghum bicolor*), dicotyledons (*B. rapa* ssp. *pekinensis*, *Theobroma cacao*, *Carica papaya*, *Brassica oleracea* and *Arabidopsis thaliana*) and *Amborella trichopoda*. We investigated the expansion and contraction of gene families using CAFÉ software (v.2.1)^47^ with a probabilistic graphical model. A random birth-and-death model was used to assess gene gain or loss in gene families across the specified phylogenetic tree. Families with P < 0.001 were considered as significant expansion or contraction, and pathway enrichment analysis of these families was conducted using the enrichment pipeline. Phylogenetic relationship among these 9 plant species was resolved using the MAFFT^43^ and FastTree package^44^, based on the 533 high-quality single-copy orthologous genes. Divergence times were estimated by the program r8s^48^, based on known divergence time between *Brassica* and *Arabidopsis* (about 20 MYA).

### 4.2 Analysis of genome synteny and whole-genome duplication

Whole-genome duplication (WGD) events were performed by searching for collinearity with the pakchoi genome using MCScan v0.8 software^49^. Repeated gene pairs located in internal collinear segments were processed for sequence alignment analysis. In addition, MCscan was also used to examine collinearity between pakchoi and Chinese cabbage, pakchoi and *A. thaliana*, Chinese cabbage and *A. thaliana*. We calculated Ks values of gene pairs within the collinear segment using synonymous_calculation (https://github.com/tanghaibao/bio-pipeline/tree/master/synonymous_calculation) based Nei-Gojobori method. The Ks distribution plot was visualized by ggplot2^50^.

### Section S5- The expression bias analysis of three subgenome homologs

### 5.1 Relative expression levels of three subgenome homologs across triads and definition of homolog expression bias categories

According to the method of R. H. Ramírez-González^51^, we focused exclusively on the gene triads which had a 1:1:1 correspondence across the three homologous subgenomes, including 1,581 syntenic (total of 4,743 genes). We defined a triad as expressed when the sum of the LF, MF1, and MF2 subgenome homologs was > 0.5 FPKM. Using this criterion, a total of 3,591 genes (1,197 triads) were defined considered expressed. To standardize the relative expression of each homolog across the triad, we normalized the absolute FPKM for each gene within the triad as follows,

$${Expression}_{LF}=\frac{FPKM(LF)}{FPKM\left( MF1 \right)+FPKM(MF2)}$$

$${Expression}_{MF1}=\frac{FPKM(MF1)}{FPKM\left( LF \right)+FPKM(MF2)}$$

$${Expression}_{MF2}=\frac{FPKM(MF2)}{FPKM\left( LF \right)+FPKM(MF1)}$$

We defined a dominant triad which only contains a single expressed homolog. The values of the relative contributions of each sub-genome per triad were used to plot the ternary diagrams using the R package ggtern^52^.

The ideal normalized expression bias for the seven categories was defined as shown in **Fig 3.** We calculated the Euclidean distance from the observed normalized expression of each triad to each of the seven ideal categories listed above. We assigned the homolog expression bias category for each triad by selecting the shortest distance.

### Section S6- RNA-seq analysis

The high-quality RNA was separately extracted from five tissues of PC-fu bolting stages, with three biological replicates. Libraries were constructed according to the protocol for the Illumina HiSeq2500 platform. We first filtered the raw RNA sequencing reads to remove low-quality bases, adaptors, duplications and potential contaminations using fastp software^53^. The remaining clean reads were then mapped onto the pakchoi genome using HISAT2 with default settings^18^. The gene expression level was quantified by FPKM, which was calculated using Cufflinks based on the gene annotation file of pakchoi genes with default parameters^54^. Expression patterns were visualized using R language.

### Section S7-BSA-seq experiment

### 7.1 Material preparation

For the BSA-seq sequencing, the glaucous varieties (JP28) and glossy varieties (JP1202) were used as the parents to generate the F_1_ and F_2_ populations. Epicuticular wax deposition was very obviously noticeable on the surface of leaf and petiole in glaucous varieties (JP28). Fewer wax was observed on surfaces of glossy varieties leaf and petiole relative to surfaces of glossy varieties (JP1202) leaf and petiole (**Fig. S10A-C**). Scanning electron microscopy (SEM) revealed a clear reduction of wax crystals in leaf and petiole surface of JP1202 (**Fig. S10D**). We developed F_2_ population by crossing JP28 and JP1202. The materials were cultivated in a Baisha farm at the Fujian Jinpin Agricultural Technology Co., Ltd in October in 2018. After flowering, population separation was analyzed, and the data were recorded.

We constructed four bulked pools, two fifty F_2_ with extreme waxy trait (S1 pool), fifty F_2_ with extreme no-waxy trait (S2 pool) and two parents pools (S3 and S4 pools), they were executed on whole-genome resequencing with 50-fold of depths.

### 7.2 BSA-seq analysis

According to the locating results of clean reads among the reference genome, duplicate reads were removed using the Picard tool (http://sourceforge.net/projects/picard/). GATK software (v4.1.4.1)^55^ was used to perform the local realignment and base recalibration to ensure the accuracy of the SNP detecting. The SNP loci between the test samples and reference genome were obtained using the GATK software according to the best practices on the GATK website (https://www.broadinstitute.org/gatk/guide/best-practices.php). All the SNP loci between the test samples were summarized according to the alignment results of test samples and the reference genome.

SNP-index were calculated to identify the candidate regions of the genome associated with waxy. The SNP-index association analysis is a method used to calculate genotype frequency differences between two pools. A SNP-index is the proportion of reads harboring the SNP that are different from the reference sequence. The Δ (SNP-index) of each locus was calculated by subtraction of the SNP-index of the S1-pool from that of the S2-pool. SNP-index = 0 if the entire short reads contain genomic fragments from S1; SNP-index = 1 if all the short reads were from S2. The average of the SNP-index was calculated in a 1 Mb interval using a sliding window analysis with 1 kb. The SNP-index graphs of the S3-pool, S4-pool and corresponding △(SNP-index) graphs were showed by a map of Manhattan (**Fig. S11; Fig 4A**).

The △(SNP-index), △(InDel-index) and △(All-index) of each marker loci in each window were counted by sliding window method. Taking △ (all index) as an example, 1000 permutation tests were conducted, and 95% confidence level was selected as the screening threshold. The black broken line in the Manhattan plot is the △(All-index) distribution in the form of window, and the window above the confidence level is taken as the candidate interval. At the 95% confidence level, the window larger than the threshold is selected as the candidate interval. In order not to ignore the influence of minor QTLs, candidate SNPs and InDels were selected in the whole genome. If the reference parent and the offspring representative type were the same, the sites with All-index close to 0 in the progeny pool were selected; if the reference parent and the offspring representative type were opposite, the candidate sites with All-index close to 1 were selected as candidate sites. ANNOVAR^56^ was annotated for the candidate polymorphic markers and the annotation results were extracted.

### Section S8- Full-length transcriptome sequencing and analysis

### 8.1 Material preparation

PC-fu (tolerant to high-temperature varieties) and JP20 (sensitive to high-temperature varieties) were selected for the temperature stress experiment. The leaves of JP20 were curled and wilted and PC-Fu leaves were slightly curled under 40°C treatment for 24 h after sowing 3-weeks (**Fig. S15**). PC-fu and JP20 sowed in soil and grown for 3 weeks at the Biotron, maintaining 22°C, and 16 h day/8 h night. Leaves were harvest and pooled for each sampling time (0 h, 12 h and 24 h under 40°C high temperature treatment).

### 8.2 Full-length transcriptome sequencing and analysis under heat stress

Full-length transcriptome data reported in this paper were obtained using Nanopore sequencing. Firstly, Raw reads were filtered with minimum average read quality score=7 and minimum read length=500bp. Ribosomal RNA were discarded after mapping to rRNA database. Next, full-length, non-chemiric (FLNC) transcripts were determined by searching for primer at both ends of reads. Clusters of FLNC transcripts were obtained after mapping to the reference genome with mimimap2^57^, and consensus isoforms were obtained after polishing within each cluster by pinfish. Consensus sequences were then mapped to the reference genome using minimap2. Mapped reads were further collapsed by cDNA_Cupcake package with min-coverage=85% and min-identity=90%. 5’ difference was not considered when collapsing redundant transcripts (https://github.com/Magdoll/cDNA_Cupcake/wiki).

For quantification of gene/transcript expression levels and differential expression analysis, full length reads were mapped to the reference transcriptome sequence. Expression levels were estimated by reads per gene/transcript per 10,000 reads mapped. Differential expression genes (DEGs) analysis was performed using the DESeq package (1.10.1)^58^. DESeq provide statistical routines for determining differential expression in digital gene/transcript expression data using a model based on the negative binomial distribution. The resulting *P* values were adjusted using the Benjamini and Hochberg’s approach for controlling the false discovery rate. Genes/transcripts with FDR < 0.01 and fold change ≥ 2 found by DESeq were assigned as DEGs. Gene expression pattern analysis of tolerance/sensitivity pakchoi were respectively perform by Short Time-series Expression Miner software (STEM)^59^ in the OmicShare tools platform (www.omicshare.com/tools). The parameters were set as follows: 1) Maximum Unit Change in model profiles between time points is 1; 2) Maximum output profiles number is 8; 3) Minimum ratio of fold change of DEGs was no less than 2.0. The significance of the mode of co-regulation of the two materials was obtained by T-test (*P-*value <0.01).

For different alternative splicing (DAS) and different transcript usage (DTU) analysis, genes/transcripts with significant DAS or DTU had at least two contrast groups with adjusted FDR < 0.01 and with these contrast groups having at least ≥10% change in PSI. To determine whether an AS event generated an isoform that contains premature stop codons and could be degraded by nonsense-mediated mRNA decay (NMD), we predicted the longest Open Reading Frame (ORF) of each isoform by TransDecoder (https://github.com/TransDecoder/TransDecoder). If the stop codon of isoform is >50 nt upstream of an exon-exon junction, the alternative splicing (AS) event was regarded as producing an NMD candidate.

### 8.3 The validation of differential AS events by RT-qPCR

cDNA was synthesized using a PrimeScript RT reagent kit with gDNA Eraser (Vazyme). RT-qPCR reactions were done using Vazyme SYBR Premix Ex Taq (Tli RNaseH Plus) on a Mastercycler ep Realplex (Eppendorf). The relative expression data was calculated by the ΔΔCt (cycle threshold) method. AS events were validated by RT-qPCR analysis, according to previous reports^60^. The inclusion/exclusion ratio was determined from the level of exon inclusion RNA normalized to the level of exon exclusion RNA. The data of the splicing efficiency were validated from three biological repeats.

PCR primers were designed to ensure that either spliced RNA or unspliced RNA was amplified. The unspliced primer pair was designed to exactly span the retained intron or alternative exon region for alternative exon/intron inclusive transcripts, and the spliced primer pair were designed to span the exon-exon junction region connecting the upstream flanking exon and the downstream flanking exon of alternative exon/intron exclusive transcripts. Sequences for primer pairs are listed in **Table S19**.

## Reference

1 Allen, G. C., Flores-Vergara, M. A., Krasynanski, S., Kumar, S. & Thompson, W. F. A modified protocol for rapid DNA isolation from plant tissues using cetyltrimethylammonium bromide. *Nature Protocols* **1**, 2320-2325 (2006).

2 Koren, S. *et al.* Canu: scalable and accurate long-read assembly via adaptive *k-mer* weighting and repeat separation. *Genome research* **27**, 722-736 (2017).

3 Hancock, J. M. *BLAT (BLAST-like Alignment Tool)*. (American Cancer Society, 2004).

4 Walker, B. J. *et al.* Pilon: An integrated tool for comprehensive microbial variant detection and genome assembly improvement. *PLOS ONE* **9**, e112963 (2014).

5 Rao, S. S. *et al.* A 3D map of the human genome at kilobase resolution reveals principles of chromatin looping. *Cell* **159**, 1665-1680 (2014).

6 Servant, N. *et al.* HiC-Pro: an optimized and flexible pipeline for Hi-C data processing. *Genome Biology* **16**, 259 (2015).

7 Li, H. & Durbin, R. Fast and accurate short read alignment with Burrows-Wheeler transform. *Bioinformatics* **25**, 1754-1760 (2009).

8 Burton *et al.* Chromosome-scale scaffolding of *de novo* genome assemblies based on chromatin interactions. *Nature Biotechnology* **31**, 1119-25 (2013).

9 Parra, G., Bradnam, K. & Korf, I. CEGMA: a pipeline to accurately annotate core genes in eukaryotic genomes. *Bioinformatics* **23**, 1061-1067 (2007).

10 Simão, F. A., Waterhouse, R. M., Ioannidis, P., Kriventseva, E. V. & Zdobnov, E. M. BUSCO: assessing genome assembly and annotation completeness with single-copy orthologs. *Bioinformatics* **31**, 3210-3212 (2015).

11 Burge, C. Prediction of complete gene structures in human genomic DNA. *Journal of Molecular Biology* **268**, 78-94 (1997).

12 Stanke, M. & Waack, S. Gene prediction with a hidden Markov model and a new intron submodel. *Bioinformatics* **19 Suppl 2**, ii215-225 (2003).

13 Majoros, W. H., Pertea, M. & Salzberg, S. L. TigrScan and GlimmerHMM: two open source ab initio eukaryotic gene-finders. *Bioinformatics* **20**, 2878-2879 (2004).

14 Blanco, E., Parra, G. & Guigó, R. Using geneid to identify genes. *Curr Protoc Bioinformatics* **Chapter 4**, Unit 4.3 (2007).

15 Korf, I. Gene finding in novel genomes. *BMC Bioinformatics* **5**, 59 (2004).

16 Keilwagen, J. *et al.* Using intron position conservation for homology-based gene prediction. *Nucleic Acids Res* **44**, e89 (2016).

17 Keilwagen, J., Hartung, F., Paulini, M., Twardziok, S. O. & Grau, J. Combining RNA-seq data and homology-based gene prediction for plants, animals and fungi. *BMC Bioinformatics* **19**, 189 (2018).

18 Kim, D., Langmead, B. & Salzberg, S. L. HISAT: a fast spliced aligner with low memory requirements. *Nat Methods* **12**, 357-360 (2015).

19 Pertea, M. *et al.* StringTie enables improved reconstruction of a transcriptome from RNA-seq reads. *Nat Biotechnol* **33**, 290-295 (2015).

20 Tang, S., Lomsadze, A. & Borodovsky, M. Identification of protein coding regions in RNA transcripts. *Nucleic Acids Res* **43**, e78 (2015).

21 Campbell, M. A., Haas, B. J., Hamilton, J. P., Mount, S. M. & Buell, C. R. Comprehensive analysis of alternative splicing in rice and comparative analyses with Arabidopsis. *BMC Genomics* **7**, 327 (2006).

22 Haas, B. J. *et al.* Automated eukaryotic gene structure annotation using EVidenceModeler and the Program to Assemble Spliced Alignments. *Genome Biol* **9**, R7 (2008).

23 Marchler-Bauer, A. *et al.* CDD: a Conserved Domain Database for the functional annotation of proteins. *Nucleic Acids Res* **39**, D225-229 (2011).

24 Koonin, E. V. *et al.* A comprehensive evolutionary classification of proteins encoded in complete eukaryotic genomes. *Genome Biol* **5**, R7 (2004).

25 Ogata, H. *et al.* KEGG: Kyoto Encyclopedia of Genes and Genomes. *Nucleic Acids Res* **27**, 29-34 (1999).

26 Boeckmann, B. *et al.* The SWISS-PROT protein knowledgebase and its supplement TrEMBL in 2003. *Nucleic Acids Res* **31**, 365-370 (2003).

27 Altschul, S. F., Gish, W., Miller, W., Myers, E. W. & Lipman, D. J. Basic local alignment search tool. *J Mol Biol* **215**, 403-410 (1990).

28 Dimmer, E. C. *et al.* The UniProt-GO annotation database in 2011. *Nucleic Acids Res* **40**, D565-570 (2012).

29 Harris, M. A. *et al.* The Gene Ontology (GO) database and informatics resource. *Nucleic Acids Res* **32**, D258-261 (2004).

30 Conesa, A. *et al.* Blast2GO: a universal tool for annotation, visualization and analysis in functional genomics research. *Bioinformatics* **21**, 3674-3676 (2005).

31 Lowe, T. M. & Eddy, S. R. tRNAscan-SE: a program for improved detection of transfer RNA genes in genomic sequence. *Nucleic Acids Res* **25**, 955-964 (1997).

32 Griffiths-Jones, S. *et al.* Rfam: annotating non-coding RNAs in complete genomes. *Nucleic Acids Res* **33**, D121-124 (2005).

33 Zheng, Y. *et al.* iTAK: A program for genome-wide prediction and classification of plant transcription factors, transcriptional regulators, and protein kinases. *Mol Plant* **9**, 1667-1670 (2016).

34 Letunic, I. & Bork, P. 20 years of the SMART protein domain annotation resource. *Nucleic Acids Res* **46**, D493-d496 (2018).

35 Prakash, A., Jeffryes, M., Bateman, A. & Finn, R. D. The HMMER web server for protein sequence similarity search. *Curr Protoc Bioinformatics* **60**, 3.15.11-13.15.23 (2017).

36 Finn, R. D. *et al.* Pfam: the protein families database. *Nucleic Acids Res* **42**, D222-230 (2014).

37 Tarailo-Graovac, M. & Chen, N. Using RepeatMasker to identify repetitive elements in genomic sequences. *Curr Protoc Bioinformatics* **Chapter 4**, Unit 4.10 (2009).

38 Claire, H. *et al.* PASTEC: An automatic transposable element classification Tool. *Plos One* **9**, e91929 (2014).

39 Zhang, Y. *et al.* Model-based analysis of ChIP-Seq (MACS). *Genome Biol* **9**, R137 (2008).

40 Ellinghaus, D., Kurtz, S. & Willhoeft, U. LTRharvest, an efficient and flexible software for de novo detection of LTR retrotransposons. *BMC Bioinformatics* **9**, 18 (2008).

41 Xu, Z. & Wang, H. LTR_FINDER: an efficient tool for the prediction of full-length LTR retrotransposons. *Nucleic Acids Res* **35**, W265-268 (2007).

42 Ou, S. & Jiang, N. LTR_retriever: A highly accurate and sensitive program for identification of long terminal repeat retrotransposons. *Plant Physiol* **176**, 1410-1422 (2018).

43 Katoh, K. & Standley, D. M. MAFFT multiple sequence alignment software version 7: improvements in performance and usability. *Mol Biol Evol* **30**, 772-780 (2013).

44 Price, M. N., Dehal, P. S. & Arkin, A. P. FastTree: computing large minimum evolution trees with profiles instead of a distance matrix. *Mol Biol Evol* **26**, 1641-1650 (2009).

45 Yu, G., Lam, T. T., Zhu, H. & Guan, Y. Two methods for mapping and visualizing associated data on phylogeny using Ggtree. *Mol Biol Evol* **35**, 3041-3043 (2018).

46 Emms, D. M. & Kelly, S. OrthoFinder: phylogenetic orthology inference for comparative genomics. *Genome Biol* **20**, 238 (2019).

47 Han, M. V., Thomas, G. W., Lugo-Martinez, J. & Hahn, M. W. Estimating gene gain and loss rates in the presence of error in genome assembly and annotation using CAFE 3. *Mol Biol Evol* **30**, 1987-1997 (2013).

48 Sanderson, M. J. r8s: inferring absolute rates of molecular evolution and divergence times in the absence of a molecular clock. *Bioinformatics* **19**, 301-302 (2003).

49 Tang, H. *et al.* Synteny and collinearity in plant genomes. *Science* **320**, 486-488 (2008).

50 Ito, K. & Murphy, D. Application of ggplot2 to Pharmacometric Graphics. *CPT Pharmacometrics Syst Pharmacol* **2**, e79 (2013).

51 Ramírez-González, R. H. *et al.* The transcriptional landscape of polyploid wheat. *Science* **361**, eaar6089 (2018).

52 Hamilton, N. & Ferry, M. ggtern : Ternary diagrams using ggplot2. *Journal of statistical software* **87**, 1 (2018).

53 Chen, S., Zhou, Y., Chen, Y. & Gu, J. fastp: an ultra-fast all-in-one FASTQ preprocessor. *Bioinformatics* **34**, i884-i890 (2018).

54 Trapnell, C. *et al.* Differential gene and transcript expression analysis of RNA-seq experiments with TopHat and Cufflinks. *Nat Protoc* **7**, 562-578 (2012).

55 McKenna, A. *et al.* The Genome Analysis Toolkit: a MapReduce framework for analyzing next-generation DNA sequencing data. *Genome Res* **20**, 1297-1303 (2010).

56 Wang, K., Li, M. & Hakonarson, H. ANNOVAR: functional annotation of genetic variants from high-throughput sequencing data. *Nucleic Acids Res* **38**, e164 (2010).

57 Li, H. Minimap2: pairwise alignment for nucleotide sequences. *Bioinformatics* **34**, 3094-3100 (2018).

58 Anders, S. & Huber, W. Differential expression analysis for sequence count data. *Genome Biol* **11**, R106 (2010).

59 Ernst, J. & Bar-Joseph, Z. STEM: a tool for the analysis of short time series gene expression data. *BMC Bioinformatics* **7**, 191 (2006).

60 Yan, Q., Xia, X., Sun, Z. & Fang, Y. Depletion of Arabidopsis SC35 and SC35-like serine/arginine-rich proteins affects the transcription and splicing of a subset of genes. *PLoS Genet* **13**, e1006663 (2017).
